# The global diffusion of artificial intelligence exposes an efficiency–transformation gap

**DOI:** 10.1016/j.ese.2026.100784

**Published:** 2026-10-29

**Authors:** Meiling Wu, Wei Liu, Ziqiong He, Zhengyang Zhang

**Affiliations:** a School of Management Science, Chengdu University of Technology, Chengdu, 610059, China; b School of Economics and Management, Nanchang University, Nanchang, Jiangxi, 330031, China; c Graduate School of Environmental Studies, Tohoku University, Sendai, 980-8572, Japan

**Keywords:** Artificial intelligence, Sustainable development goals (SDGs), Efficiency-transformation gap, Technological diffusion, Climate governance

## Abstract

Global development agendas increasingly rely on artificial intelligence to accelerate sustainability transitions. As automated systems integrate into power grids and industrial operations, policymakers assume these technological upgrades will naturally translate into broader ecological gains. Yet it remains unclear whether algorithmic efficiency drives the deep structural changes needed for global climate action, or merely optimizes the status quo. Here we show an inherent efficiency–transformation gap in how artificial intelligence affects global sustainability. Analyzing 69 countries from 2000 to 2023, we find that technological diffusion robustly improves operational capacities—such as clean energy access (SDG 7) and industrial infrastructure (SDG 9)—driven by measurable gains in human capital and reduced national energy intensity. However, this techno-efficiency does not extend to systemic environmental governance. Artificial intelligence yields no robust progress in responsible consumption (SDG 12) or climate action (SDG 13). Instead, the rapid deployment of digital infrastructure temporarily crowds out green innovation, exposing a short-term friction between operational optimization and ecological transition. Furthermore, scenario projections to 2100 indicate that even under highly optimistic sustainability pathways, technological growth will not automatically erase regional development divides. Realizing the full promise of artificial intelligence requires policies that explicitly align computational power with structural climate action, rather than relying on efficiency gains to solve ecological crises.

## Highlights

• AI diffusion robustly advances global infrastructure and energy access but fails to drive systemic climate action.
• These operational gains stem directly from enhanced human capital and a measurable drop in national energy intensity.
• Expanding digital capabilities temporarily crowds out green innovation, creating friction in systemic ecological governance.
• Scenario projections to 2100 show that technological optimization alone cannot close structural global development divides.

## Graphical abstract

**Figure:**
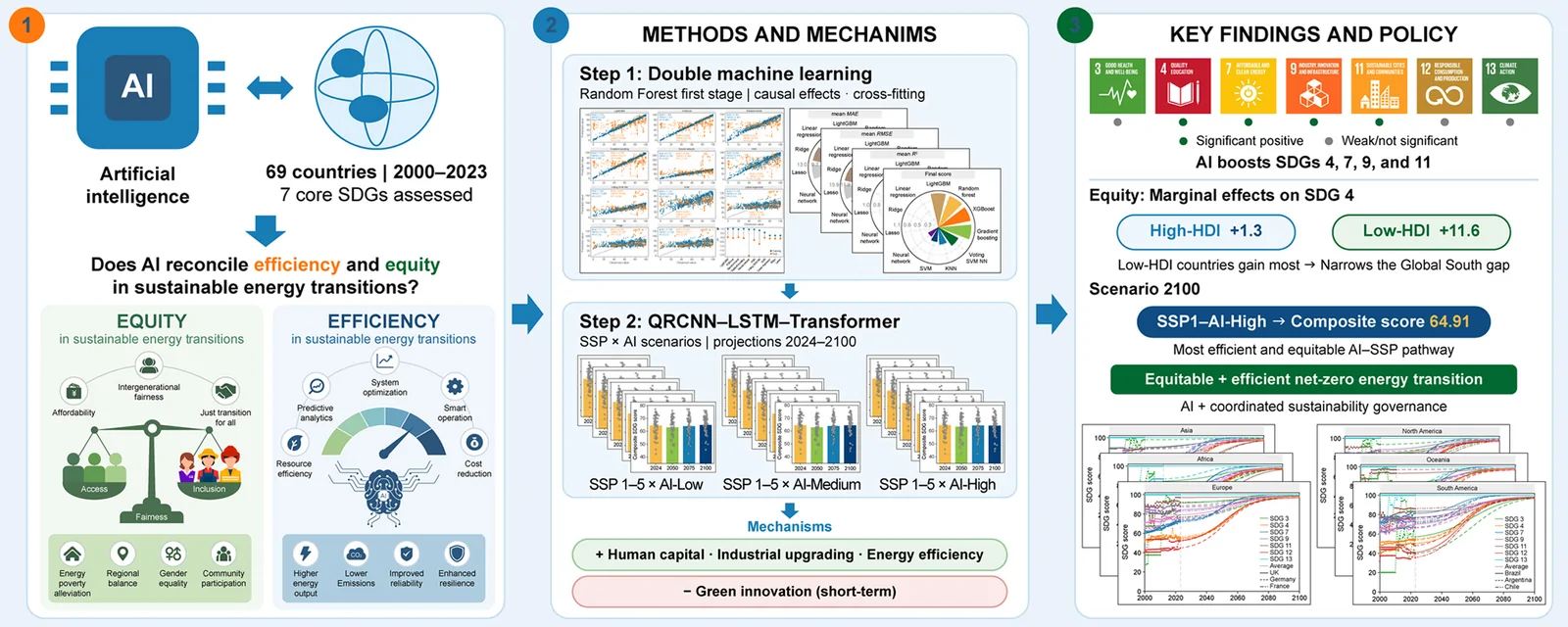

## 1 Introduction

Artificial intelligence (AI) is increasingly integrated into the systems that underpin sustainable development, including energy supply [1], industrial production, urban governance, healthcare, education services, and environmental monitoring [2], [3]. It has become an important enabling capacity through which countries coordinate energy use, allocate social resources, deliver public services, and manage environmental risks, with direct implications for sustainable development in areas such as health and well-being, quality education, clean energy access, industrial innovation, sustainable urban development, resource use, and climate governance [4]. However, as the 2030 target for meeting the global Sustainable Development Goals (SDGs) draws closer, progress across these areas remains marked by substantial cross-country and sectoral disparities [5]. Persistent deficits in clean energy access, low-carbon industrial upgrading, public service provision, resource efficiency, and climate adaptation indicate that technological progress does not necessarily translate into development progress [6]. The key empirical issue is therefore whether AI diffusion has generated measurable sustainability gains, and whether those gains extend beyond countries with strong technological and institutional capacity.

Although AI can improve efficiency across energy, industrial, environmental, and public service systems, its contribution to sustainable development remains conditional. AI can reduce forecasting errors in electricity systems, support the integration and consumption of renewable energy, optimize industrial production, strengthen environmental regulation, and improve the allocation of healthcare and education resources [7], [8]. These benefits, however, depend on computing infrastructure, reliable electricity supply, high-quality data, skilled labor, and regulatory capacity, which are unevenly distributed across countries [9]. The expansion of AI-related industries may also increase electricity demand, intensify critical mineral consumption, accelerate hardware replacement, and widen existing digital divides. In sustainable energy transitions [10], [11], AI-enabled efficiency gains may therefore coexist with technological dependence and unequal development opportunities [12]. A cross-country empirical assessment is needed to clarify how AI affects different sustainability outcomes, how these effects vary by development level, and whether AI can help reconcile efficiency and equity in sustainability transitions.

Recent literature increasingly frames AI as a general-purpose digital technology that may reshape the pathways countries pursue for sustainable development and energy transition. In this study, AI refers to algorithmic techniques and to the broader socio-technical capacity formed by data infrastructure, computing resources, digital skills, innovation systems, and governance arrangements [13], [14]. Sustainable development, in turn, denotes the coordinated advancement of economic prosperity, social inclusion, resource efficiency, and environmental protection, while the SDGs provide a globally comparable framework for translating this broad development agenda into measurable outcomes [15]. Within this framework, sustainable energy transition is central because SDG 7 is closely connected with climate action, industrial innovation, responsible consumption, health, poverty reduction, and social equity [16]. Existing studies show that technological innovation and energy access can advance SDG progress by improving productivity, expanding renewable energy, supporting green entrepreneurship, and strengthening policy capacity [17]. However, infrastructure, financing, institutional quality, trade linkages, and development conditions often mediate these effects, suggesting that the energy transition should be evaluated by aggregate environmental performance and by its implications for efficiency and equity [18].

Recent studies have begun to link AI and sustainable development by highlighting its potential to improve resource allocation, environmental governance, and low-carbon transformation. AI can enhance energy demand forecasting, smart grid management, renewable energy integration, industrial energy efficiency, pollution monitoring, and green finance allocation, thereby creating new opportunities for more efficient sustainability transitions. Empirical evidence shows that AI may reduce climate risk through resource efficiency, green innovation, more effective environmental policy, and green finance [19]. Other studies have found that AI can improve green productivity by promoting renewable energy use, attracting skilled labor, and enhancing the efficiency of economic and environmental systems [13]. Research on urban sustainability further suggests that AI can reduce emissions by improving production efficiency, lowering energy consumption, promoting green technological innovation, facilitating industrial upgrading, and fostering public participation [20]. These findings suggest that AI can become an important enabler of sustainable development, especially when digital capabilities are effectively integrated with energy, industrial, and environmental systems [17], [21], [22].

Nevertheless, AI's sustainability implications remain conditional and uneven, making an SDG-based assessment particularly important. AI systems require substantial electricity, computing infrastructure, data processing capacity, specialized human capital, and institutional support. These prerequisites are unevenly distributed across countries and regions, potentially creating new forms of environmental and developmental inequality. Recent evidence shows that AI may amplify carbon inequality due to its energy requirements and unequal deployment capacity. However, stronger trade openness, greater participation in global value chains, stronger government capacity, and strict institutional transparency can mitigate these adverse effects [23]. Other studies report nonlinear, threshold-dependent effects, indicating that AI-related sustainability gains are more likely to materialize when renewable energy capacity, human capital, infrastructure, and governance systems are sufficiently developed [24]. AI literature on fulfilling SDG goals also suggests that AI is associated with multiple development goals rather than a single environmental outcome [25]. For this study, the most relevant SDG domains include clean energy, industrial innovation and infrastructure, inequality reduction, responsible production and consumption, and climate action. These core SDGs jointly capture the key sustainability dimensions in which AI may reshape energy-related development outcomes, including technological efficiency, energy accessibility, resource optimization, distributive fairness, and climate compatibility.

Existing research on energy transition and SDGs further demonstrates that clean energy development is both a technical and a social process. Studies on renewable energy, hydrogen, energy access, and environmental technological innovation show that energy transition can generate synergies across clean energy, industrial upgrading, climate action, poverty reduction, health, and resource efficiency [26], [27]. However, these studies also highlight trade-offs associated with infrastructure costs, policy coordination, financial capacity, regional inequality, and access to technology. Evidence from the Global South indicates that energy access projects contribute to SDG 7 and broader development objectives only when they are connected to local needs, inclusive governance, and sustainable socioeconomic outcomes [28], [29]. Research on green hydrogen and renewable energy innovation similarly suggests that low-carbon technologies may support multiple SDGs. Nevertheless, high upfront costs, weak infrastructure, and insufficient policy support constrain their benefits [30]. These insights are directly relevant to the AI-enabled energy transition: AI may improve energy-system efficiency, but its contribution to SDG-related outcomes depends on whether countries have the complementary capacities needed to translate digital technologies into equitable and sustainable development gains.

Despite growing research on AI-enabled sustainability and increasing attention to SDGs, a critical gap persists in understanding whether AI diffusion is associated with balanced progress across core SDG domains, rather than gains concentrated mainly in efficiency-oriented outcomes [31]. Existing studies have provided valuable evidence of AI's role in carbon reduction, pollution control, climate-risk mitigation, green productivity, environmental governance, energy transition, and broader SDG progress [32], [33]. However, these studies often rely on single-dimension environmental indicators or aggregate sustainability indices and have not sufficiently examined how AI may affect specific SDG domains in uneven, context-dependent ways [34], [35]. Cross-country empirical evidence, therefore, remains limited on whether AI diffusion is consistently associated with human development, clean energy, industrial innovation, sustainable cities, responsible production and consumption, climate action, and distributive outcomes. AI-driven advances are more directly linked to resource allocation, energy optimization, industrial upgrading, and environmental monitoring, whereas their implications for distributive equity, inclusive access, and cross-country development gaps remain less well understood [34], [36]. Examining these differentiated relationships is therefore necessary to understand how AI can be aligned with inclusive, efficient, and sustainable development.

Here, we investigate the relationship between AI development and progress towards selected Sustainable Development Goals (SDGs) from a global perspective, focusing on seven SDGs spanning health, education, energy, innovation, urban sustainability, responsible consumption and climate action [37]. This study examines how AI development and selected SDG outcomes have evolved globally; how AI diffusion is associated with different SDG domains, including human development, clean energy, innovation, sustainable cities, responsible consumption, and climate action; and which future AI–socioeconomic pathways may support progress across these SDGs. To address these questions, we first construct a multi-country assessment of AI development and core SDG performance. We then apply double machine learning (DML) to identify differentiated relationships and potential mechanisms between AI diffusion and selected SDG outcomes while accounting for high-dimensional controls, nonlinear relationships, and potential confounding factors. Finally, we develop an integrated quantile regression, convolutional neural network, and long short-term memory (QRCNN–LSTM) transformer forecasting framework under alternative shared socioeconomic pathways (SSPs) to project future SDG trajectories. By combining machine-learning-based empirical analysis with scenario-oriented forecasting, this study provides an integrated assessment of the relationship between AI development and SDG progress across different development contexts.

## 2 Methods and materials

### 2.1 Research methods

#### 2.1.1 Double machine learning estimation

Estimating the effect of AI development on SDG performance requires disentangling AI's contribution from the broader socioeconomic and institutional conditions under which AI technologies emerge, diffuse, and operate. In a cross-country setting, AI development is closely associated with income level, human capital, innovation capacity, industrial structure, energy use, environmental pressure, and governance quality. These factors may simultaneously affect SDG outcomes, producing substantial confounding in the observational data. Moreover, these relationships are unlikely to be strictly linear, and correlations among national development indicators may further weaken the reliability of estimates from a fully parametric specification. The empirical strategy, therefore, needs to accommodate nonlinear confounder adjustment while retaining a clearly interpretable parameter for the AI–SDG relationship.

The DML estimator proposed by Chernozhukov et al. [38] is particularly suitable for research settings in which the parameter of primary interest is low-dimensional, while the observed confounders may exhibit complex nonlinear or high-dimensional associations. This framework is appropriate for estimating the relationship between AI development and SDG performance after controlling for multiple interrelated national characteristics. Compared with conventional regression-based approaches, DML does not require specifying relationships between the control variables, the treatment, and the outcome through a fixed linear functional form. Instead, these nuisance components are estimated flexibly using machine learning algorithms. Unlike purely predictive machine learning methods, DML retains an explicit inferential target parameter, allowing the estimated AI effect to remain interpretable. Through Neyman orthogonality and cross-fitting, the estimator further reduces sensitivity to nuisance-function estimation errors, overfitting, and regularization bias [39]. Therefore, DML was used as the baseline estimator in this study.

Following the partially linear DML framework, the outcome and treatment equations are specified as:

(1) Y=βT+g(X)+ε

(2) T=m(X)+v

Here, Y is the score of the core SDG under analysis and T is the AI development level. X is the vector of eight observed national control variables; its component $X_{j}$ is the j th control variable, with j ranging from 1 to 8. The scalar β is the adjusted AI–SDG effect parameter of interest. The function g(X) maps the controls to the structural outcome component, and m(X) maps the controls to the conditional mean of AI development. The disturbances ε and v are the outcome and treatment errors, respectively; both have conditional mean zero given X.

The expectation operator is denoted by E. The conditional mean of SDG performance given the control variables is defined as g (X) = E [Y |X], and the conditional mean of AI development is m (X) = E [T |X]. Under equations (1), (2), the conditional outcome mean satisfies g (X) = βm (X)+ f (X). This relation distinguishes the conditional outcome mean from the structural outcome component.

The estimation procedure first obtains machine learning predictions of both SDG performance and AI development conditional on the selected control variables. The predicted components are then removed from the original outcome and treatment variables to construct residualized variables. The target parameter is estimated from the association between the residualized SDG score and the residualized AI development level. Formally, the orthogonal moment condition is expressed as:

(3) E[{(Y−g(X)−β[T−m(X)]}[T−m(X)]]=0

Provided that the residual variation in AI development is nonzero, the target parameter can equivalently be expressed as:

(4) $$\beta =\frac{E\left[\left(Y-g\left(X\right)\right)\left(T-m\left(X\right)\right)\right]}{E\left[{\left(T-m\left(X\right)\right)}^{2}\right]}$$

In practice, machine learning predicts the conditional means of SDG performance and AI development from the selected controls, and cross-fitting enables orthogonalized estimation. The resulting coefficient β represents the estimated adjusted effect of AI development on each core SDG score after accounting for the observed socioeconomic, technological, environmental, and institutional covariates.

#### 2.1.2 Scenario setting

To evaluate the long-term influence of AI development on core SDGs under different socioeconomic conditions, we combined five SSPs (SSP1–SSP5) [40], [41] with three AI-development trajectories (AI-high, AI-medium, and AI-low), generating fifteen SSP–AI scenario combinations for 2025–2060. This coupled scenario design suits the present study because future SDG performance depends on the pace of AI diffusion and on the broader socioeconomic conditions under which AI is developed and applied. A single baseline projection or a scenario framework that considers only socioeconomic pathways would be insufficient to capture this joint uncertainty. By integrating SSP-based socioeconomic futures with alternative AI development trajectories, the proposed scenario setting enables examination of the long-term AI–SDG relationship across heterogeneous development contexts and provides a more targeted framework for assessing AI's role in sustainable development.

The socioeconomic dimension follows the widely used SSP framework proposed by O'Neill et al. [42] and Riahi et al. [43], including SSP1 (sustainability), SSP2 (middle of the road), SSP3 (regional rivalry), SSP4 (inequality), and SSP5 (fossil-fueled development). Each SSP defines the evolution of five exogenous drivers (i.e., gross domestic product, urbanization rate, population, education level, and government efficiency) used to govern the dynamic simulation of SDG trajectories.

Because the standard SSP framework does not explicitly characterize future AI development, we introduced three AI development trajectories according to relative growth rates: AI-high (1.6), representing rapid technological progress supported by strong infrastructure, investment, and innovation capacity; AI-medium (1.0), serving as the baseline trajectory that maintains historical growth trends; and AI-low (0.6), representing slower AI diffusion under weak policy support, inadequate infrastructure, or constrained research and development (R&D) capacity. Combining SSP1–SSP5 with these three AI trajectories creates a two-dimensional scenario framework and extends conventional SSP-based projections by directly incorporating variation in the key explanatory factor of interest. Detailed parameter values are provided in Supplementary Table S1.

#### 2.1.3 QRCNN–LSTM–transformer

We developed an integrated QRCNN–LSTM–transformer model to generate scenario-based, long-term SDG projections for SSP–AI combinations. This hybrid architecture captures the nonlinear, nonstationary, multiscale relationships among socioeconomic development, AI evolution, and SDG outcomes. In the proposed framework, the QRCNN module extracts local, nonstationary temporal features; the LSTM component captures sequential dependencies across historical observations; and the transformer module models broader temporal and scenario-related dependencies [44]. By combining these complementary functions, the model projects SDG trajectories under multiple future scenarios [45].

We trained and validated the model using historical data from 2000 to 2023. Observations from 2000 to 2018 were used for model training, whereas observations from 2019 to 2023 were used for validation. The optimization process first compared alternative look-back window lengths and then tuned the network structure and training hyperparameters. We evaluated candidate look-back windows of 4, 5, 6, and 7 years using validation metrics, including *RMSE*, *MAE*, and *MAPE*, and retained the five-year look-back window because it delivered the best overall validation performance and balanced short-term information extraction with medium-term temporal-dependency capture. In this setting, the look-back window refers to the length of the historical input sequence used in each one-step-ahead prediction. The model uses the preceding five annual observations of the relevant SDG and explanatory variables to predict the next period's SDG outcome. This does not mean that the model was trained using only five years of data or that the trajectories to 2100 were directly extrapolated from a single five-year sequence.

After determining the optimal look-back window and model parameters, the trained model generated long-term SDG projections for 2024–2100 under different SSP–AI scenario combinations. In this stage, we incorporated scenario-specific trajectories of socioeconomic and AI-related drivers into the input sequences and projected SDG outcomes through a recursive, year-by-year procedure. For each projection step, the model used the most recent five-year sequence, updated with newly generated outputs and the corresponding scenario inputs, to produce the next annual SDG value. The final network structure consisted of a 1D convolutional (Conv1D) layer with 32 filters and a kernel size of 2, followed by MaxPooling1D, two LSTM layers with 64 and 32 units, a transformer encoder with four attention heads and a feed-forward dimension of 64, and a linear dense output layer. We trained the model with the Nadam optimizer at a learning rate of 0.001, a batch size of 8, and 100 training epochs, with early stopping to reduce overfitting. No dropout was used in the final configuration. Supplementary Text S1 provides detailed descriptions of the look-back window optimization, structural parameter selection, training procedure, validation results, and recursive scenario projection strategy.

### 2.2 Variable descriptions

This study constructs a cross-country panel dataset to examine the relationship between AI development and core SDG performance. The empirical framework includes three groups of variables: AI development level as the key explanatory variable; selected core SDG scores as outcome variables; and socioeconomic, technological, environmental, and institutional factors as control variables. This design enables us to assess whether countries with stronger AI capacity show different sustainable development performance after accounting for major national background conditions. All variables were collected from internationally recognized databases and official institutional sources. Supplementary Table S2–S4 report detailed definitions, computational formulas, units, indicator attributes, and official database links.

#### 2.2.1 AI development level

The key explanatory variable is the AI development level, which measures a country's overall ability to generate, absorb, and apply AI-related technologies. It captures the national AI ecosystem across technological innovation, industrial deployment, scientific knowledge production, digital infrastructure, human capital, and frontier technology readiness [46], [47]. In the empirical analysis, this variable serves as the treatment variable in the DML framework, as the study aims to estimate the adjusted relationship between national AI capacity and sustainable development performance. Theoretically, AI may affect SDG outcomes through productivity improvements, industrial upgrading, energy system optimization, environmental monitoring, public service delivery, and more efficient resource allocation [48].

Following previous studies [49], [50], we constructed the AI development level index from six positive indicators: AI-related patents, industrial robot stock, AI-related scientific publications, secure internet servers per million people, R&D technical personnel per million people, and Information and Communications Technology (ICT) frontier readiness. These indicators reflect innovation capability, industrial application, knowledge creation, digital infrastructure, human capital support, and technological readiness, respectively, thereby providing a multidimensional measure of AI development rather than relying on a single proxy.

The AI indicators were obtained from the World Intellectual Property Organization (WIPO), the International Federation of Robotics (IFR), the World Bank, the United Nations Educational, Scientific and Cultural Organization (UNESCO) Institute for Statistics, and the World Economic Forum. We standardized all indicators to remove scale differences, then aggregated them using the entropy method to construct a comparable country–year index. Detailed indicator definitions, formulas, sources, and official links are provided in Supplementary Table S3; the standardization and aggregation procedures are described in Supplementary Text S2.

#### 2.2.2 Core SDGs

The outcome variables are the performance scores of seven core SDGs: SDG 3 (Good Health and Well-being), SDG 4 (Quality Education), SDG 7 (Affordable and Clean Energy), SDG 9 (Industry, Innovation, and Infrastructure), SDG 11 (Sustainable Cities and Communities), SDG 12 (Responsible Consumption and Production), and SDG 13 (Climate Action) [4], [17], [51]. These SDGs were selected to represent key sustainability domains in which AI may have direct or indirect effects, including health resource allocation, digital education, energy efficiency, industrial innovation, smart city governance, resource management, and climate risk monitoring [52], [53].

For each SDG, secondary indicators were compiled in accordance with the United Nations (UN) SDG indicator framework revised in 2023 [54]. We obtained data mainly from the UN SDG Global Database (https://unstats.un.org/sdgs/), supplemented as needed by the World Health Organization (WHO), UNESCO, the World Bank, International Energy Agency (IEA), UN-Habitat, United Nations Environment Programme (UNEP), Organisation for Economic Co-operation and Development (OECD), United Nations Framework Convention on Climate Change (UNFCCC), and the Global Carbon Atlas. We standardized positive and negative indicators by contribution direction and calculated composite SDG scores using the entropy method. Indicator codes, units, attributes, sources, and official links are listed in Supplementary Table S4; calculation procedures are provided in Supplementary Text S2.

#### 2.2.3 Control variables

To reduce potential omitted variable bias when estimating the relationship between AI development and core SDG performance [55], [56], we included eight categories of control variables in the empirical model. These form the control vector X in the DML framework because they may simultaneously influence both AI development and SDG outcomes. Controlling for these factors helps separate the association between AI and SDG performance from broader differences in national development conditions. Specifically, GDP per capita controls for economic development and technology-adoption capacity; total population captures demographic scale and pressure on public services and infrastructure; R&D expenditure reflects innovation intensity; gross enrolment ratio represents education and human capital; manufacturing value added captures industrial structure; clean energy share reflects energy transition; per-capita CO_2_ emissions measure environmental pressure; and government efficiency captures institutional quality and policy implementation capability.

The variables were obtained from the World Bank's World Development Indicators, the UNESCO Institute for Statistics, the International Energy Agency, the Global Carbon Atlas, and the Worldwide Governance Indicators. Detailed definitions, code names, data sources, and official database links are provided in Supplementary Table S2.

### 2.3 Sample selection and representativeness

This study examines the relationship between AI and sustainable development. From a theoretical perspective, AI's effects on sustainable development likely vary across countries because of differences in technological foundations, development stages, institutional contexts, and resource allocation capacity. To capture this heterogeneity, the study includes countries with complete, comparable data on AI indicators, SDG indicators, and key socioeconomic control variables for 2000–2023, while excluding those with substantial missing data, inconsistent statistical definitions, or limited comparability across core variables. The final sample comprises 69 countries, spanning economies with diverse income levels, human development stages, technological capacities, and regional characteristics. Therefore, the sample is not based on arbitrary or convenience selection but reflects a balance between theoretical relevance, data completeness, and global representativeness, providing an appropriate basis for examining the heterogeneous effects, transmission mechanisms, and scenario evolution of AI on sustainable development.

## 3 Results

### 3.1 Global AI disparities and alignment with SDGs

Between 2000 and 2023, the global mean AI development index was 0.2 (standard deviation = 0.3), ranging from 0 to 1, indicating substantial disparities in AI adoption among countries (Supplementary Table S5). This variation reflects pronounced gaps in technological infrastructure and imbalances in policy investment and innovation environments. Among the seven core SDGs, the highest mean scores were observed for SDG 9 (78.7), SDG 11 (78.0), and SDG 13 (75.6), followed by SDG 3 (61.0) and SDG 7 (63.2), whereas SDG 4 (49.4) and SDG 12 (41.2) showed comparatively lower performance. Variation among countries also differed across SDGs, with SDG 13 exhibiting the largest standard deviation (s.d. = 30.7), reflecting uneven progress in climate governance.

Classification of countries into seven AI–SDG categories (A1–A7) showed that countries with higher AI development levels generally exhibited higher SDG scores, whereas countries with intermediate AI levels showed greater variation in SDG performance (Fig. 1). Countries with high AI levels tended to cluster toward the upper range of SDG scores, while those in the mid-range AI group exhibited a wider spread and more extreme values. Most countries were at intermediate stages of AI development, but SDG performance varied: SDGs 4 and 12 remained relatively low, whereas SDGs 3, 7, 9, 11, and 13 were concentrated in the medium-to-high range, indicating an overall upward trend. SDG 9 displayed a relatively stable distribution across AI levels.

**Fig. 1:**
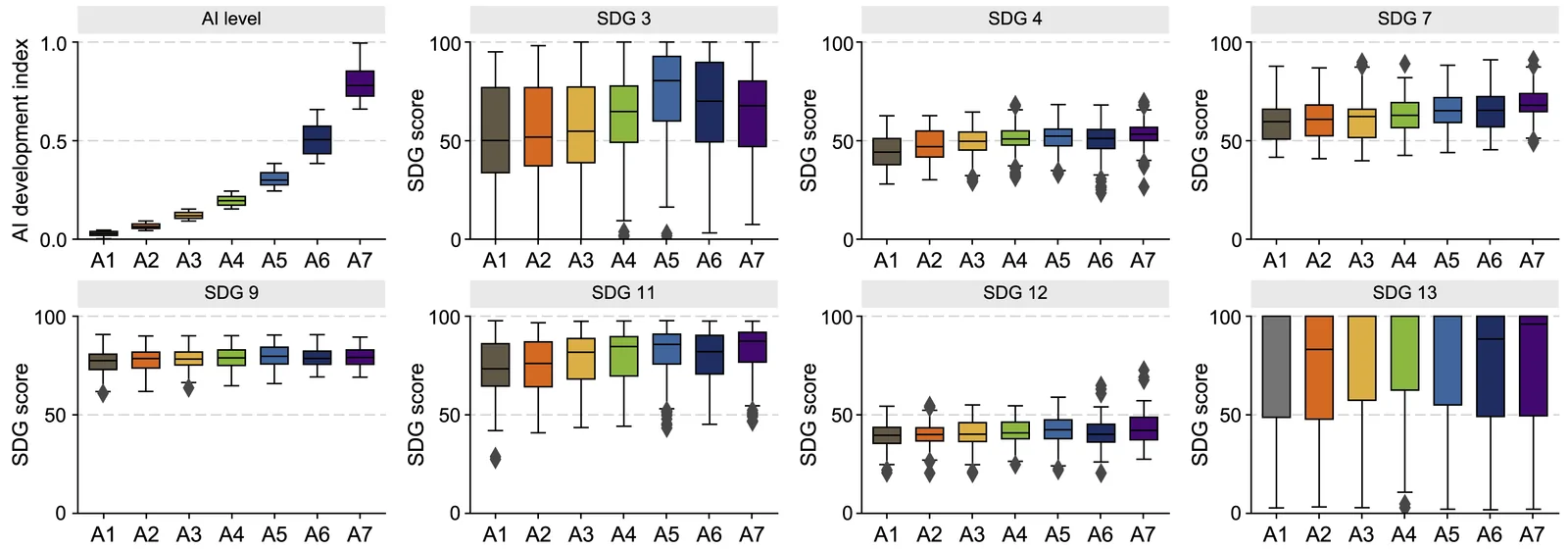
Distributions of AI development and core SDG scores across seven quantile groups, 2000–2023. Box plots show the artificial intelligence (AI) index and scores for Sustainable Development Goals (SDGs) 3, 4, 7, 9, 11, 12, and 13. A1–A7 denote seven quantile groups defined separately for each variable, ordered from the lowest (A1) to the highest (A7) values. Each group contains approximately the same number of observations, but its value range and country membership may differ among variables. Boxes indicate interquartile ranges (IQR), horizontal lines denote medians, and whiskers show 1.5 × IQR of the lower and upper quartiles. Points beyond the whiskers indicate outliers.

Overall, AI development and SDG performance exhibited a positive gradient relationship, with SDGs 9 and 11 most strongly aligned with AI levels. Persistent gaps in education and responsible consumption underscore the need to explore mechanisms that link AI and sustainable development outcomes.

### 3.2 Causal effects of AI on core SDGs

Before estimating the net effect of AI development on each core SDG, we assessed the correlation structure among the treatment variable, outcome variables, and control variables to evaluate the suitability of the selected variables and identify potential estimation risks associated with including a relatively rich set of controls. The treatment variable, AI, did not show problematic dependence on the outcome variables or the control set that would invalidate subsequent estimation (Fig. 2). However, after introducing multiple country-level socioeconomic and environmental controls, we observed relatively strong pairwise correlations among some controls in specific regions. In Africa, X6 (energy structure) and X8 (government efficiency) showed a high negative correlation (*r* = −0.9). These patterns did not indicate severe collinearity in the overall model, but suggested potential multicollinearity risks in parts of the control set when conventional regression methods were applied [57].

**Fig. 2:**
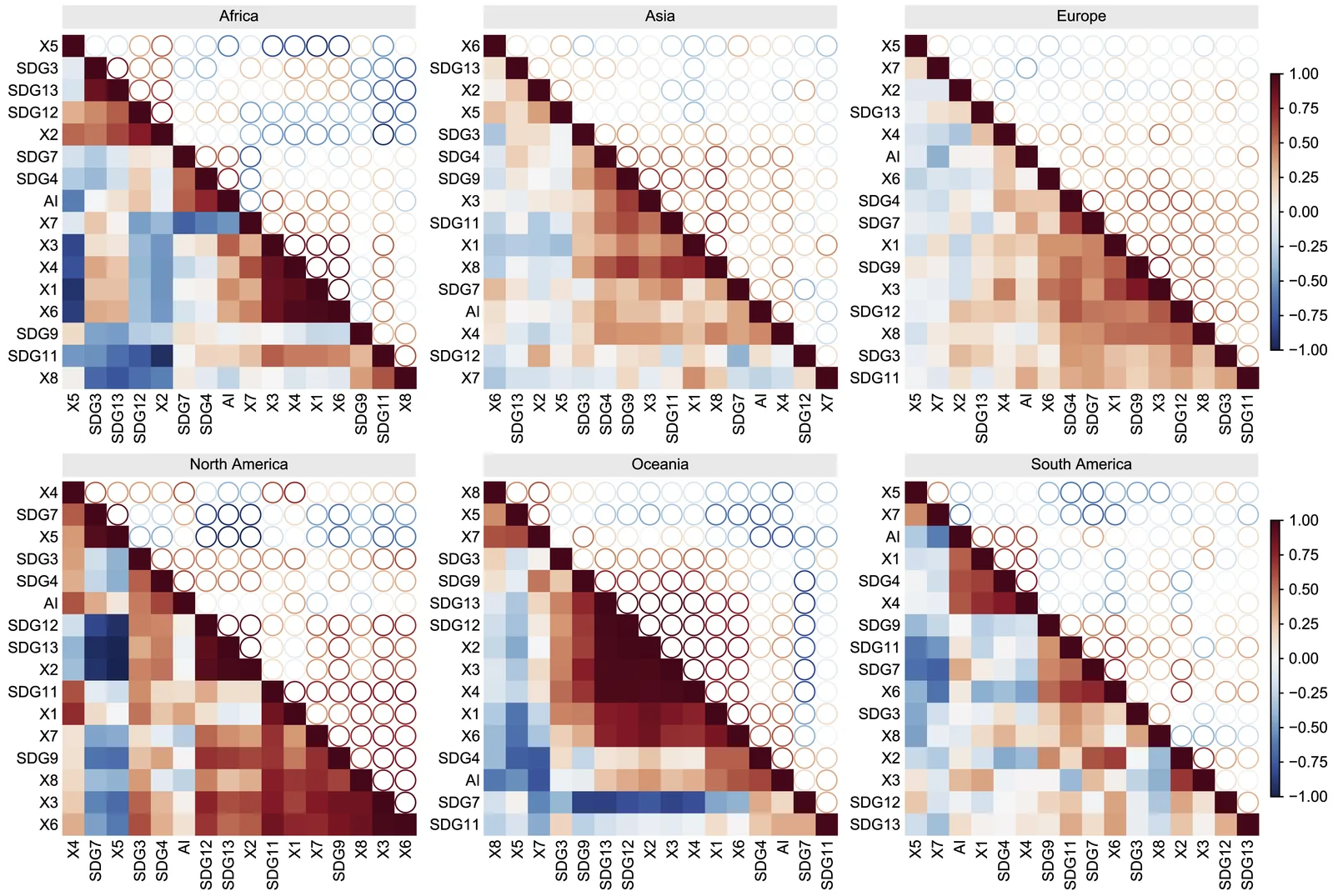
Regional correlation matrices of AI, core SDGs, and control variables. The regions include Africa, Asia, Europe, North America, Oceania, and South America. Colors indicate the direction and magnitude of Pearson's correlation coefficients according to the shared scale. The lower and upper triangles display the coefficients using colored cells and outlined symbols, respectively. Variables are ordered by hierarchical clustering using average linkage on a distance matrix defined as 1 − *r*, where r is the Pearson correlation coefficient. Clustering is performed separately for each region, and the dendrogram leaf order is used to reorder the variables. AI, artificial intelligence; SDG, Sustainable Development Goal; X1, economic development; X2, population scale; X3, innovation intensity; X4, education; X5, industrial structure; X6, energy structure; X7, environmental pressure; X8, government efficiency. Data sources and calculation details for X1–X8 are provided in Supplementary Table S2.

Given this empirical setting, we used the DML framework to estimate the associations between AI and SDG performance. DML is suitable for identifying treatment effects after conditioning on relatively high-dimensional controls because it flexibly partials out confounding influences through machine-learning-based nuisance estimation and cross-fitting. In the present analysis, this framework helped reduce potential estimation instability associated with localized correlations among control variables while retaining the net contribution of AI.

This study compared multiple first-stage learners within the DML procedure because nuisance-function estimation quality directly affects the reliability of final treatment effect estimates. In this multi-outcome setting, we evaluated 11 supervised learning models, including Light Gradient Boosting Machine (LightGBM), Extreme Gradient Boosting (XGBoost), random forest, gradient boosting, neural network, k-nearest neighbors (KNN), voting, support vector machine (SVM), linear regression, ridge, and least absolute shrinkage and selection operator (Lasso), using *MAE*, *RMSE*, and *R*^2^ (Supplementary Table S6). All models used the same predictor set and a unified preprocessing pipeline, including standardizing all predictors.

Tree-based models substantially outperform linear models and support vector regression, K-nearest neighbors, and neural networks (Fig. 3 and Supplementary Table S7). Light GBM ranked first in integrated score and achieved the lowest average prediction errors (mean *MAE* = 6.1, mean *RMSE* = 8.4), while XGBoost had the highest average explanatory power (mean *R*^*2*^ = 0.6). Random forest ranked second overall (final score = 0.97), with mean *MAE* of 6.3, mean *RMSE* of 8.6, and mean *R*^*2*^ of 0.6, showing predictive performance comparable to LightGBM, XGBoost, and Gradient Boosting. Random forest exhibited the lowest cross-task variability in prediction errors, with the smallest standard deviation of *MAE* = 4.5 and standard deviation of *RMSE* = 6.1, as well as a relatively low standard deviation of *R*^*2*^ = 0.2, indicating a more stable generalization across SDG outcomes. Based on this comparison, we selected random forest as the benchmark first-stage learner in the DML framework. We chose this not because random forest was the best model across all predictive metrics, but because it provided an effective balance of predictive accuracy, cross-task stability, and methodological robustness. Random forest can flexibly capture nonlinear relationships and higher-order interactions, is less sensitive to hyperparameter tuning, and delivers stable, competitive predictive performance across multiple SDG tasks. Therefore, we used it as the primary first-stage learner in the main analysis, while LightGBM and XGBoost, two other high-performing tree-based models, served as alternative first-stage learners in robustness checks to assess whether the estimated treatment effects were sensitive to the choice of machine learning algorithm.

**Fig. 3:**
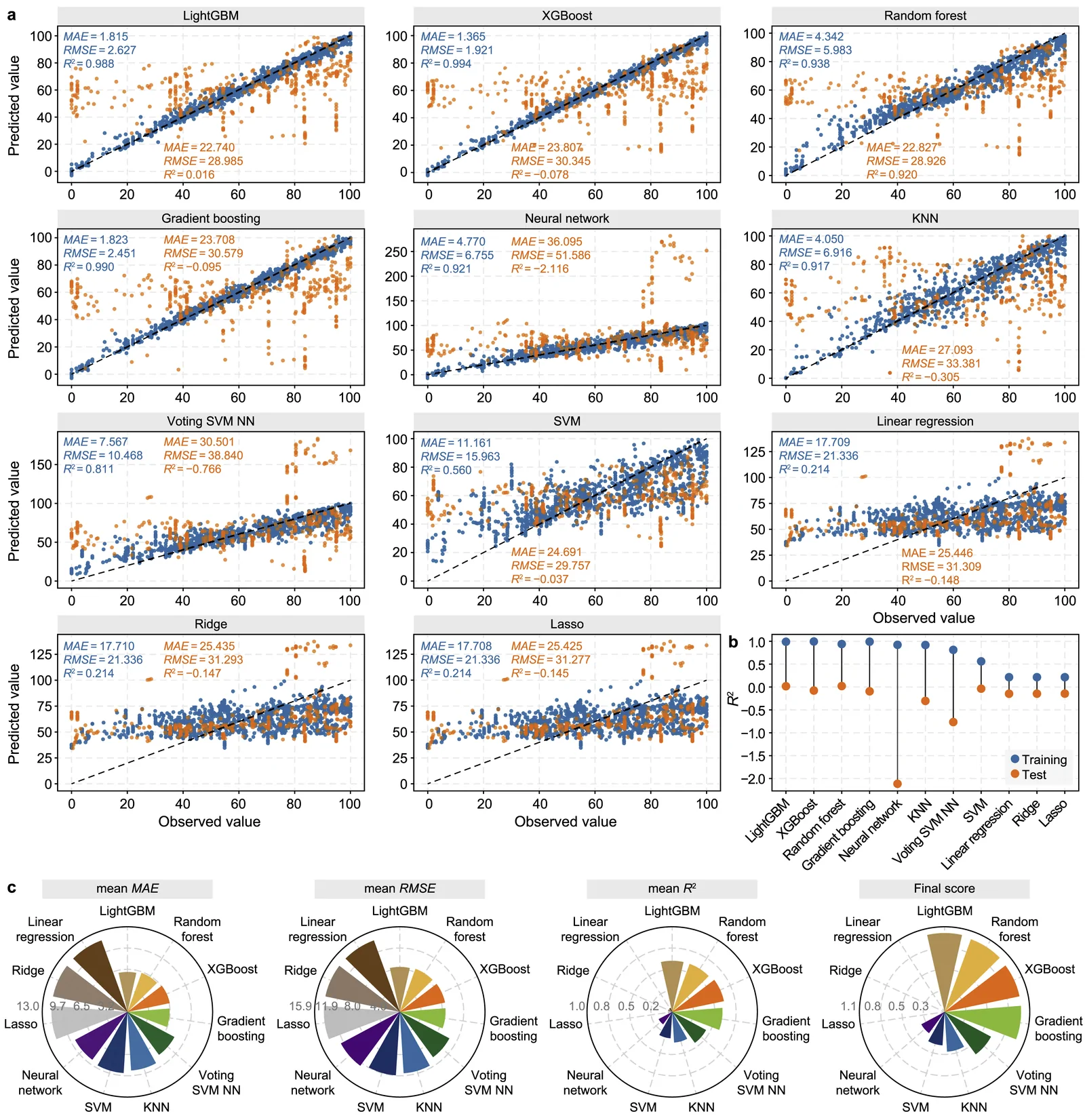
Predictive performance of candidate first-stage learners. **a**, Predicted versus observed values for 11 candidate learning algorithms in the training and test sets. Dashed diagonal lines indicate perfect agreement. Blue and orange circles denote training and test data points, respectively. The 11 models are Light Gradient Boosting Machine (LightGBM), Extreme Gradient Boosting (XGBoost), random forest, gradient boosting, neural network, k-nearest neighbors (KNN), voting, support vector machine (SVM), linear regression, ridge, and least absolute shrinkage and selection operator (Lasso). **b**, Training- and test-set coefficients of determination (*R*^2^) for each model. **c**, Model performance averaged across the seven SDG outcomes, evaluated using mean absolute error (*MAE*), root mean square error (*RMSE*), *R*^2^, and a composite performance score. The composite score combines normalized *R*^2^, reverse-normalized *MAE*, and reverse-normalized *RMSE*, with higher scores indicating better overall predictive performance. All models were compared using the same dataset, training–test split, preprocessing pipeline, random seed, and evaluation protocol. Model configurations are provided in Supplementary Table S6. SDG, Sustainable Development Goal.

We further evaluated the cross-fitting configuration used in the DML procedure. Cross-fitting reduces overfitting bias and improves the robustness of treatment-effect estimation by separating nuisance-function estimation from final causal estimation. We compared different fold settings before implementing the benchmark model, and a six-fold configuration provided the best balance between estimation significance and prediction error. Accordingly, we adopted a random forest model with six-fold cross-fitting in the subsequent analysis. Detailed comparisons among different cross-fitting configurations are provided in Supplementary Fig. S1, and the full model configuration information is reported in Supplementary Text S3.

The benchmark DML estimations were conducted under four model specifications that gradually incorporated control variables, country fixed effects, and time fixed effects [55] (Supplementary Table S8). In the baseline specification with country fixed effects only, AI showed significant positive associations with SDGs 3, 4, 7, 9, 12, and 13, with particularly large coefficients for SDG 3 and SDG 11. After adding time fixed effects, the coefficients for SDG 3 and SDG 11 increased markedly, indicating that the estimated contribution of AI became more evident after accounting for unobserved temporal heterogeneity.

After introducing control variables, the positive effects of AI on SDGs 4, 7, 9, and 11 remained significant, although their magnitudes declined, suggesting that country-level structural factors absorbed some of the explanatory power. In the full specification, which included all controls and country- and time-fixed effects, AI continued to have significant positive effects on SDGs 4, 7, 9, and 11. A moderate positive effect also re-emerged for SDG3 (*β* = 7.85, *p* < 0.05). By contrast, the effects on SDGs 12 and 13 were not statistically significant in the full specification. These results indicate that AI had the most stable positive effects on education, clean energy, industrial innovation, and sustainable urban development, while its effects on sustainable production and climate action were weaker and more heterogeneous.

The robustness checks supported the main DML findings (Supplementary Table S9). Varying the number of cross-fitting folds from the benchmark six-fold configuration to five- and seven-fold configurations produced minimal changes in coefficient signs, magnitudes, and significance levels [58], [59]. Replacing the random forest learner with LightGBM, XGBoost, and ridge regression resulted in moderate variation in some indicators, but the positive effects on SDGs 4, 7, and 9 remained stable across all learner configurations. The results for SDGs 3 and 11 differed between the linear ridge model and the nonlinear learners, suggesting nonlinear response patterns. For SDGs 12 and 13, the coefficients varied in sign and significance, consistent with the benchmark finding that AI's effects on these two goals were not robustly significant. Overall, the robustness tests confirmed that AI's positive effects were most reliable for SDGs 4, 7, and 9, with additional evidence of positive effects on SDGs 11 and 3 under the full specification.

### 3.3 Mechanisms and heterogeneity of AI impacts

To identify the channels through which AI affected sustainable development outcomes, we examined six potential mechanisms: human capital, green innovation, government efficiency, industrial structure upgrading, total factor productivity, and energy efficiency (Supplementary Table S10) [38], [60]. These mechanism variables represented two major dimensions of AI influence: technology application pathways and production efficiency pathways.

The first-stage mechanism estimations showed the heterogeneous effects of AI across the six channels (Supplementary Table S11). AI had significant positive effects on human capital (*β* = 0.010), industrial structure upgrading (0.021), and total factor productivity (0.004), suggesting that AI development was associated with improvements in skill accumulation, economic structural transformation, and productivity enhancement [61].

By contrast, AI showed significant negative effects on green innovation (*β* = −0.010) and government efficiency (*β* = −0.001). AI was associated with a significant reduction in energy intensity (*β* = −0.040, measured as energy use per USD 1000 of GDP, with lower values denoting greater energy efficiency), indicating reduced energy use per unit of GDP and improved energy efficiency. These results suggest that AI's intermediate effects were not uniformly positive across all transmission channels, with green innovation showing the most pronounced negative response [62].

The second-stage DML estimations further assessed how these mechanism variables were associated with the seven core SDGs (Supplementary Table S12). The results showed substantial heterogeneity across both mechanisms and SDG dimensions. Human capital had the strongest positive association with SDG 4, indicating that education-related sustainable development was particularly sensitive to improvements in human capital. However, its effects on some energy and industry-related goals were weaker or negative. Green innovation was positively associated with SDGs 3, 4, and 9, but it showed less favorable associations with SDGs 7 and 11. Government efficiency generally exerted positive effects across several social and economic SDG dimensions, suggesting that institutional capacity remained an important enabling condition for translating AI-related development into sustainability outcomes.

Industrial structure upgrading and total factor productivity contributed the most to SDGs 7, 9, 11, and 12, underscoring the relevance of economic transformation and productivity improvement for energy access, industrial innovation, urban sustainability, and sustainable production. Energy efficiency showed positive effects on SDGs 3 and 12, indicating that resource-use efficiency was more closely linked to health-related and circular production-related outcomes. Overall, the mechanism results suggest that AI affects SDG performance through multiple pathways, but the direction and magnitude of these indirect effects vary across both mechanisms and SDG targets [53], [63].

Heterogeneity in AI impacts was further examined across country groups classified by development status and the UN Human Development Index (HDI). Using HDI-based classifications, countries were assigned to low- and high-HDI groups according to their HDI values in the baseline year of 2000, with a UN Development Program cutoff of 0.55 [55] (Fig. 4 and Supplementary Table S13). In developed countries, AI had significant positive effects on SDGs 4 (*β* = 1.7), 7 (2.8), and 9 (0.8), whereas the effects on SDGs 3 and 13 were weak and statistically insignificant. These results suggest that in developed economies, AI contributed more consistently to education, clean energy, and industrial innovation than to health or climate-related outcomes.

**Fig. 4:**
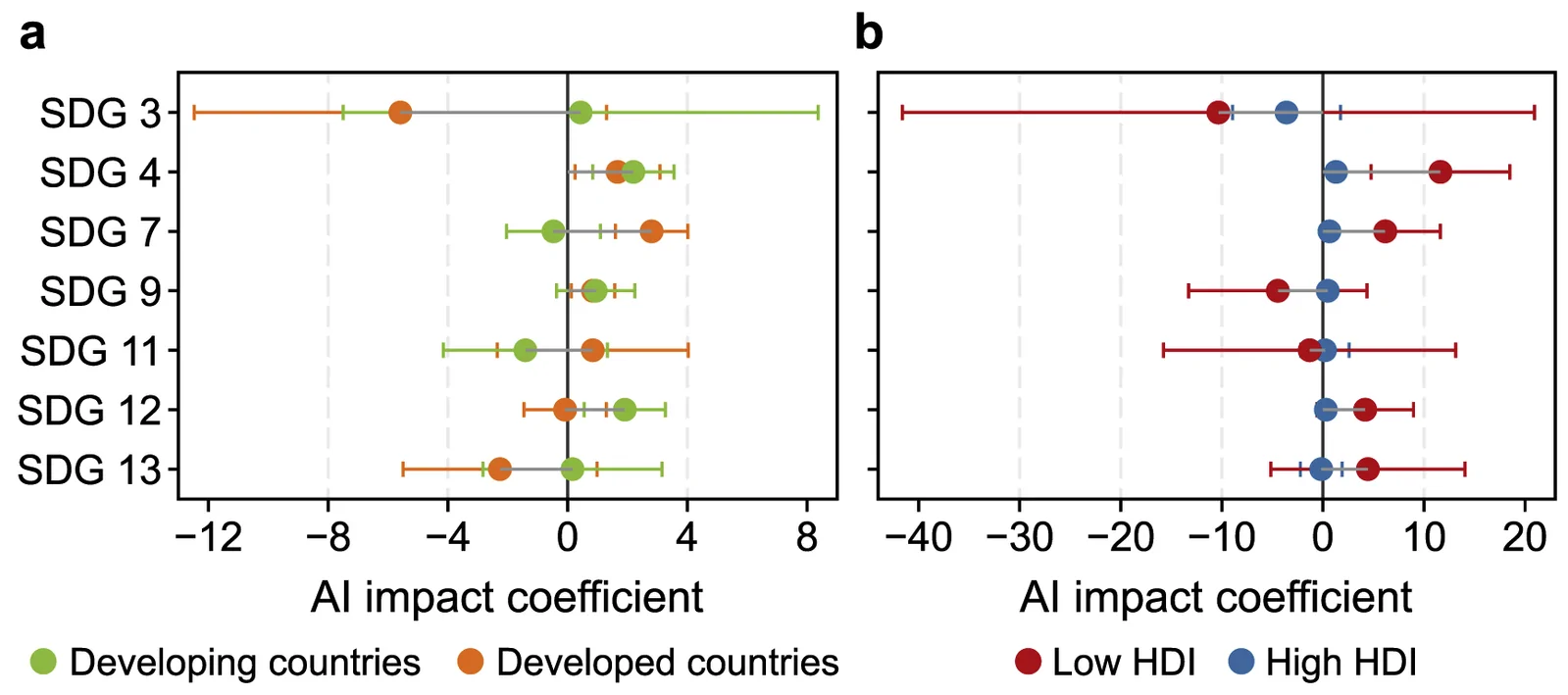
Estimated associations between AI development and SDG scores across country groups. Estimates are compared between developed and developing countries (**a**) and between high- and low-HDI countries (**b**). Points indicate estimated coefficients; all points are uniformly sized, and horizontal error bars represent 95% confidence intervals (CIs) based on DML standard errors. The vertical dashed line at zero indicates no estimated effect; the *x*-axis shows coefficient estimates (95% CI). Grey lines connect the two groups within each SDG. Developed and developing countries were classified following the International Monetary Fund (IMF) classification, fixed at the sample base year 2000. High- and low-HDI groups were defined using United Nations Development Programme (UNDP) Human Development Index (HDI) in 2000 with a threshold of 0.550. AI, artificial intelligence; SDG, Sustainable Development Goal; HDI, human development index; DML, double machine learning.

In developing countries, AI exerted stronger positive effects on SDGs 4 (*β* = 2.2) and 12 (*β* = 1.9, *p* < 0.01), while its effects on other SDGs were not statistically significant. This pattern indicates that AI-related gains in developing economies are more concentrated in education and sustainable production. Compared with developed countries, the larger coefficient for SDG 4 suggests that AI may have generated greater marginal improvements in education-related outcomes in countries with relatively less mature baseline educational resources and digital infrastructure.

The HDI-based grouping showed a similar but more pronounced pattern (Fig. 4 and Supplementary Table S13). In high-HDI countries, AI was mainly associated with improvements in SDG 4 (*β* = 1.3), whereas its associations with the other SDGs were weaker or statistically insignificant. By contrast, in low-HDI countries, AI exhibited markedly larger positive associations with SDGs 4 (*β* = 11.6), 7 (6.2), and 12 (4.2), suggesting greater potential for AI-related improvements in education, clean energy, and sustainable production in contexts with lower baseline levels of human development.

Given that the low-HDI subsample comprises only three countries (i.e., India, Morocco, and Pakistan), this study further conducted a leave-one-country-out sensitivity analysis in which the DML associations were re-estimated after sequentially excluding each country (Supplementary Table S14); the positive associations of AI with SDGs 4, 7, and 12 were preserved in sign under all specifications, indicating that no single country drove these results. The results indicate that AI's marginal effects were stronger in countries with lower human development levels, particularly in education, clean energy, and sustainable production. The comparison between high- and low-HDI groups suggests that AI impacts were not evenly distributed across development contexts, and that countries with lower baseline development conditions exhibited greater potential for AI-related SDG improvements in selected domains.

### 3.4 Future SDG pathways under AI–SSP scenarios

To evaluate the long-term implications of AI development for global sustainability, we combined the estimated AI effects and transmission pathways with SSPs [64]. This scenario framework generated 15 AI–SSP combinations based on three AI development trajectories and five SSP pathways, which were used to project future core SDG performance across 69 countries from 2024 to 2100.

Across the 15 scenario combinations, the mean composite scores of the core SDGs generally remained within a relatively narrow range of approximately 63–65 in many projected years. Scenario divergence became more pronounced over time (Fig. 5). Under high-AI trajectories, most SSP pathways maintained relatively higher SDG performance, with only moderate fluctuations. In contrast, medium- and low-AI trajectories, particularly under SSP3–SSP5, showed more pronounced declines or weaker long-term stability, indicating that slower technological development may constrain future sustainability performance under less favorable socioeconomic pathways.

**Fig. 5:**
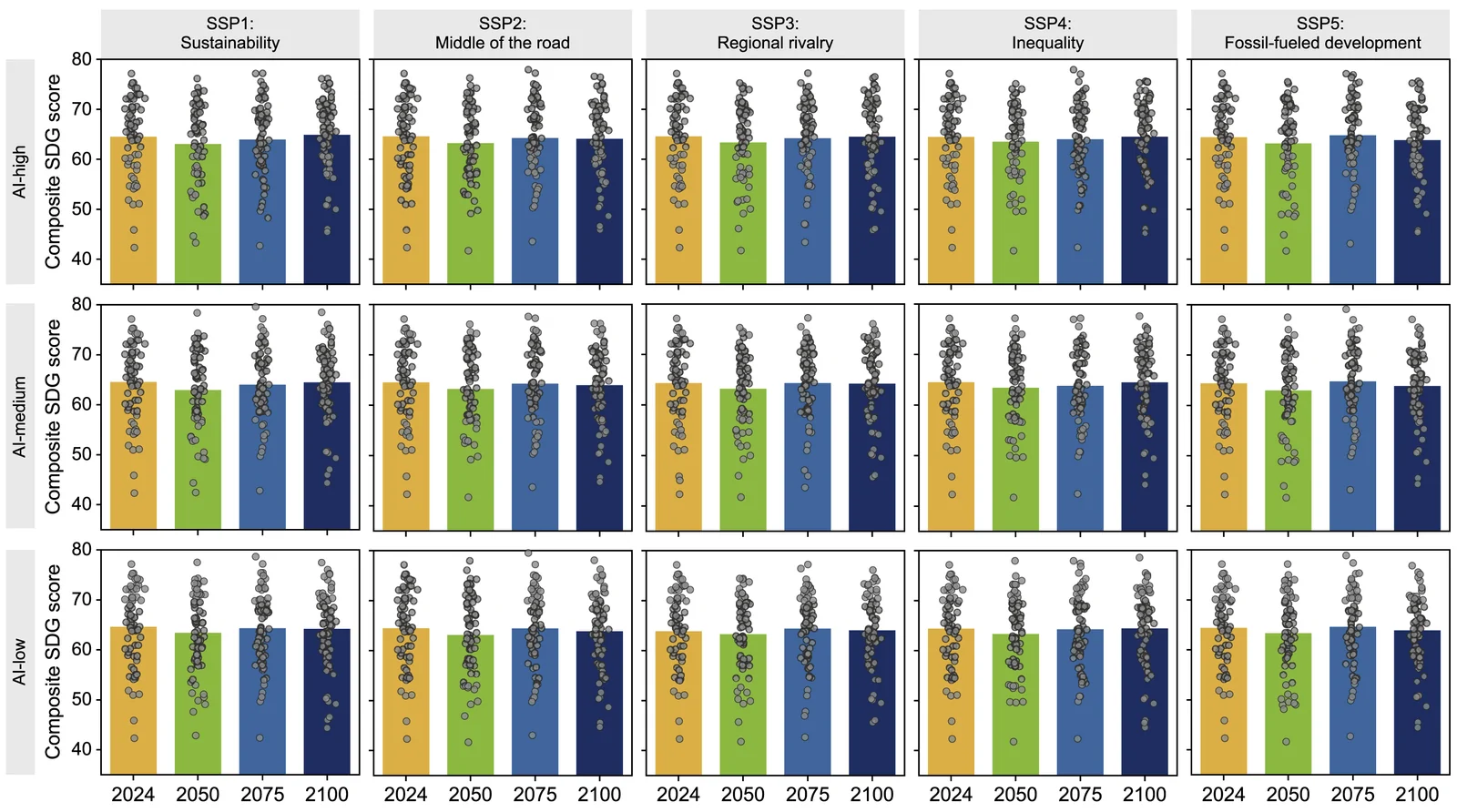
Projected distributions of composite SDG scores under 15 combinations of AI development and socioeconomic pathways. Columns represent Shared Socioeconomic Pathways (SSP1–SSP5), and rows represent high, medium, and low AI-development trajectories defined using historical growth parameters. Each panel shows projected composite scores for 69 countries in 2024, 2050, 2075, and 2100. Grey points represent individual countries, and colored bars indicate the mean score across countries. The composite score summarizes SDGs 3, 4, 7, 9, 11, 12, and 13, with indicator weights determined using the entropy weight method based on the full historical sample (2000–2023) and aggregated as a weighted sum. AI, artificial intelligence; SSP, Shared Socioeconomic Pathway.

SSP1 consistently produced the strongest SDG performance across all AI development trajectories. Under the SSP1–AI-high scenario, annual mean SDG scores remained the highest after 2025 and exceeded the second-ranked pathway by approximately 0.5–2.0 points in most projected years. The projected mean composite scores under this pathway reached 64.5 in 2024, 63.1 in 2050, 63.9 in 2075, and 64.9 in 2100. Other SSP pathways combined with high AI generally remained between 63.2 and 64.8. In SSP1, the AI-high trajectory achieved the highest projected mean score in 2100 (64.91), followed by AI-low (64.49) and AI-medium (64.47). Although differences among AI trajectories were relatively small in the early projection period, the AI-high pathway showed lower long-term dispersion across countries. These results identified SSP1–AI-high as the most favorable scenario for improving global core SDG performance.

After identifying SSP1–AI-high as the optimal scenario, we further examined its spatial and temporal patterns across regions (Fig. 6 and Supplementary Fig. S2). By 2050, the projected global distribution showed clear regional stratification. High-performing regions, including North America, Western Europe, and Oceania, maintained mean composite scores above 67.6, with Australia and Canada exceeding 72.0. Medium-level scores, ranging from 53.9 to 64.2, were mainly concentrated in Eastern Europe, Latin America, and Western Asia. Low-performing regions, including many countries in Africa, South Asia, and parts of Southeast Asia, remained below 54, indicating that substantial regional gaps persisted, even under the most favorable AI–SSP pathway.

**Fig. 6:**
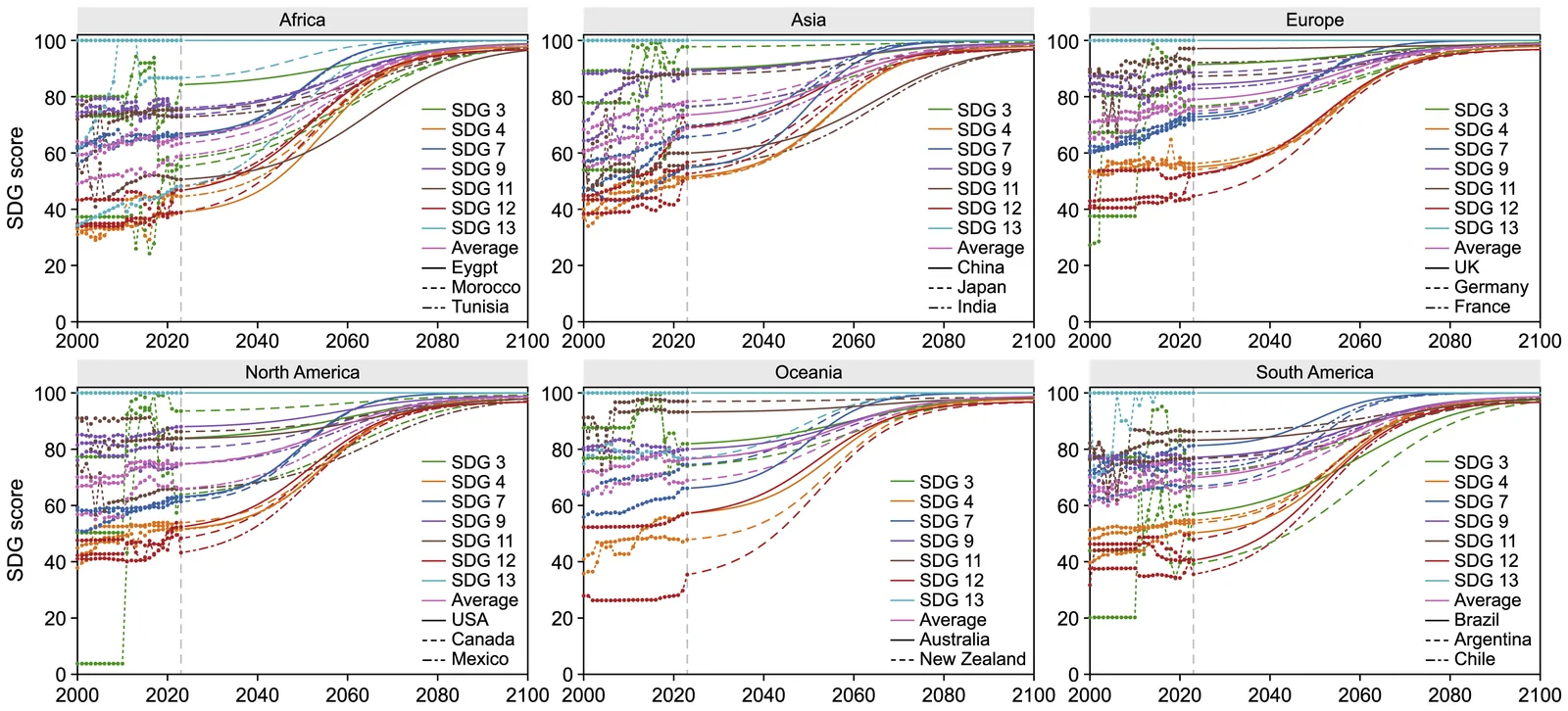
Historical and projected SDG scores for selected countries under the SSP1–AI-high scenario. Scores are shown for major regional economies in Africa, Asia, Europe, North America, Oceania, and South America over 2000–2100. Colors identify the seven SDGs and their unweighted average, and line styles distinguish countries within each region. “Average” denotes the unweighted arithmetic mean of the seven SDG scores for each country and year. Vertical dashed lines separate the historical period from the projection period beginning in 2024. Projections follow SSP1 combined with the high AI-development trajectory. Selected countries are the largest economies by GDP in 2000 within each region in the sample of 69 countries. AI, artificial intelligence; SDG, Sustainable Development Goal; SSP, Shared Socioeconomic Pathway; UK, United Kingdom; USA, United States of America.

The temporal trajectories further showed that the developed regions maintained their advantages throughout the projection period. North America and Europe remained at the upper end of the distribution, with several SDGs, including SDG 3, 7, and 9, approaching or exceeding 90 by around 2040 and rising further after 2070. Oceania gradually approached comparable performance by mid-century and showed strong outcomes in SDGs 11 and 13. Asia and South America exhibited steady upward trajectories. In Asia, China and Japan are projected to achieve high rankings in SDGs 7, 9, and 3 after 2060. South America also showed continuous improvement, although gains in SDGs 4 and 12 remained relatively moderate.

Africa displayed the largest relative improvement over the projection period. Regional scores increased from approximately 40–60 in the 2020s to levels close to the global average by 2100. Improvements were particularly evident in energy- and climate-related indicators in countries such as Egypt and Morocco. Nevertheless, the projected catch-up process occurred later than in other regions, suggesting that initial development conditions continued to shape the timing and magnitude of future SDG gains.

Overall, the scenario projections indicated that rapid AI development combined with a sustainability-oriented socioeconomic pathway was associated with improved long-term SDG performance. However, regional disparities persisted throughout the century's projections, with high-baseline regions achieving early gains, middle-baseline regions showing gradual convergence, and low-baseline regions following a later but substantial catch-up trajectory.

## 4 Discussion

### 4.1 Uneven AI effects across different SDGs

This study shows that AI diffusion is associated with sustainable development in a differentiated rather than a uniform manner. AI has stronger and more consistent associations with SDGs 4, 7, 9, and 11, suggesting it translates more readily into improvements in education, clean energy systems, industrial innovation, and urban governance. This finding is broadly consistent with existing research showing that AI and related digital technologies improve information processing, optimize resource allocation, and enhance system efficiency in data-intensive and operationally codifiable sectors [52]. In these areas, AI can often be embedded directly into service delivery, infrastructure management, and production processes, producing relatively visible gains more quickly.

However, the weaker and less stable associations with SDGs 12 and 13 indicate that AI does not automatically lead to more sustainable production patterns or stronger climate performance. This result adds nuance to the optimistic view that AI inherently supports the green transition. Although AI can improve environmental monitoring, forecasting, and operational efficiency, these gains do not necessarily reduce total material throughput, carbon dependence, or environmentally harmful production structures. Instead, such outcomes depend strongly on energy structure, regulatory incentives, and broader institutional conditions. Therefore, our findings align with studies warning that digitalization may simultaneously improve efficiency while intensifying electricity demand, data center expansion, and material consumption when low-carbon energy systems and environmental governance remain insufficient [65], [66].

This distinction is particularly important for sustainable energy transitions. AI can improve energy-system efficiency by supporting load forecasting, renewable-energy integration, grid optimization, demand-side management, and predictive maintenance. These functions help explain the relatively strong association between AI and SDG 7. However, energy transition efficiency does not necessarily imply environmental sustainability at the system level [36]. If AI applications rely on carbon-intensive electricity, expand energy-intensive computing infrastructure, or accelerate the turnover of digital devices, their net environmental benefits may be weakened. In this sense, AI may reduce inefficiencies within specific energy or industrial processes while simultaneously increasing aggregate energy and material demand.

Therefore, the uneven effects identified in this study suggest that AI contributes more significantly to the efficiency-oriented dimensions of sustainable development than to dimensions requiring deeper ecological and governance transformations. SDGs 12 and 13 require not only technological optimization but also changes in consumption behavior, production organization, regulatory enforcement, and clean-energy infrastructure. AI may support cleaner production, emissions monitoring, and renewable integration, but it cannot substitute for regulatory ambition, green investment, and behavioral change [50]. Thus, AI should not be understood as inherently sustainable. Rather, it functions as a conditional accelerator whose contribution to SDG progress depends on whether it is embedded in supportive policy, energy, and institutional environments.

These results further highlight that AI's developmental value depends on its technological capability and on the sustainability outcome under consideration [17], [29]. The key question is therefore not simply whether AI benefits sustainable development but where, how, and under what conditions AI reshapes the balance between efficiency improvement and equity protection. This perspective is especially relevant to sustainable energy transitions, where AI may improve the technical and economic efficiency of low-carbon systems while also altering the distribution of transition opportunities, capabilities, and benefits across regions and countries.

### 4.2 Mechanisms, heterogeneity, and policy implications

The mechanism analysis clarifies how AI contributes to sustainable development. Human capital accumulation, industrial upgrading, and total factor productivity are the main positive channels, indicating that AI development is primarily associated with SDG progress by strengthening knowledge capacity, restructuring economic activity, and improving production efficiency. This finding is consistent with prior literature linking AI to innovation, productivity growth, and structural transformation [67]. AI operates less as an isolated technological input than as a capability-enhancing system whose developmental effects depend on the absorptive capacity of firms, workers, and institutions.

This mechanism is particularly relevant to energy transitions. AI can enhance energy-transition efficiency only when economies possess sufficient human capital, industrial capabilities, and the organizational capacity to absorb and apply digital technologies. For example, AI-based energy management requires skilled personnel, high-quality data, interoperable digital infrastructure, and adaptive institutions [68], [69]. Without these complementary assets, AI may remain confined to isolated applications and fail to generate system-wide improvements in clean energy deployment, energy access, and energy efficiency. The positive mechanisms identified in this study indicate that AI-enabled energy transitions depend not only on algorithmic progress but also on broader socio-technical capabilities.

At the same time, the negative short-term responses of green innovation and, to a lesser extent, government efficiency indicate transitional friction in AI diffusion, despite its association with reduced energy intensity. In practice, early AI deployment often focuses on commercially profitable applications rather than environmental governance or public service optimization. Moreover, AI can generate sustainability gains only when complementary conditions are in place, including interoperable data systems, administrative capacity, regulatory adaptation, and cross-sector coordination [70], [71]. These findings imply that AI may improve productive efficiency faster than it improves ecological governance, thereby creating transitional tensions between technological upgrading and environmental sustainability. They also caution against treating AI adoption itself as sufficient evidence of sustainable transformation [72], [73].

The potentially negative externalities of AI further reinforce this caution. AI systems may increase electricity demand, expand data center infrastructure, accelerate hardware replacement, and intensify the demand for critical minerals. These pressures can offset part of the efficiency gains associated with AI, especially in regions where electricity systems remain carbon-intensive or environmental regulation is weak. In addition, AI may concentrate technological advantages among firms and countries with superior data resources, computing capacity, and institutional readiness [74]. As a result, AI can improve aggregate transition efficiency while simultaneously reproducing or widening inequalities in transition capacity. This dual effect is central to understanding how AI reshapes equity and efficiency in sustainable energy transitions.

The heterogeneity analysis further supports this interpretation. AI has stronger marginal effects in developing and low-HDI countries, particularly for SDGs 4, 7, and 12, suggesting that AI may offer leapfrogging opportunities in countries with relatively low baseline development levels [35], [74]. From a sustainable energy transition perspective, AI can help less developed economies improve energy access, optimize distributed energy systems, and accelerate the diffusion of cleaner technologies. However, this potential does not imply automatic convergence. Countries with larger marginal gains often also face greater constraints in digital infrastructure, skilled labor, electricity reliability, clean energy investment, and institutional capacity. Without corresponding investments in these foundations, AI may yield localized improvements while reinforcing broader structural inequalities within and between countries.

The scenario analysis further illustrates how AI may shape the long-term balance between transition efficiency and equity. The SSP1–AI-high pathway produces the most favorable long-term SDG outcomes, indicating that AI can substantially improve transition efficiency when technological progress aligns with a sustainability-oriented socioeconomic trajectory. On this pathway, AI is more likely to support efficient energy allocation, renewable-energy integration, demand-side management, infrastructure optimization, and more responsive public governance. However, even under this favorable pathway, persistent regional disparities show that efficiency gains do not automatically translate into equitable outcomes [75]. Regions with stronger digital infrastructure, skilled labor, clean electricity systems, and institutional capacity are better positioned to convert AI capabilities into energy transition benefits, whereas less prepared regions may experience slower diffusion and weaker gains.

This equity–efficiency tension suggests that governing AI-enabled energy transitions should go beyond technology deployment alone. AI policy should be embedded in broader sustainability governance rather than pursued as a standalone technology strategy. Policies that focus solely on accelerating AI adoption may improve short-term efficiency but fail to deliver inclusive, environmentally durable transitions [3]. More effective approaches should combine AI advancement with investments in education, digital infrastructure, clean energy, environmental regulation, and institutional capacity. For developing and low-HDI countries, policy priorities should include affordable digital infrastructure, AI-related skills, reliable electricity systems, and access to clean energy technologies. For developed countries, the policy focus should include green AI standards, low-carbon computing, responsible data governance, and reductions in the environmental footprint of digital infrastructure [3], [76].

At the international level, cooperation in technology transfer, green digital infrastructure, open scientific collaboration, and capacity building remains essential to ensure that AI-enabled energy transitions are achieved efficiently and equitably [77]. The findings suggest that AI does not automatically advance sustainable development; rather, its contribution depends on the conditions under which energy transition efficiency and equity are achieved. Whether AI contributes to balanced, sustainable transformation or creates new environmental and distributive pressures depends on governance frameworks, deployment contexts, and the distribution of technological capabilities.

### 4.3 Limitations and future work

This study has several limitations. First, cross-country indicators may not fully capture within-country differences in AI adoption, digital infrastructure, and SDG performance. Second, although the DML framework mitigates estimation bias with high-dimensional controls, causal interpretation remains conditional on the adequacy of the observed covariates and model specification. Unobserved or incompletely captured factors, including country-specific macroeconomic and geopolitical shocks, institutional factors such as corruption control, and time-varying effects associated with events such as the global financial crisis and the COVID-19 pandemic, may influence the estimated relationships. Third, long-term AI–SSP projections remain uncertain due to future technological progress, socioeconomic transitions, and policy implementation.

Future research should further assess AI's impact on individual SDGs, subtargets, and different sustainability dimensions. More detailed sectoral and subnational analyses are needed to reveal how AI affects education quality, energy transition, industrial restructuring, sustainable consumption, and climate governance in different institutional contexts. Future studies should also examine distributional effects, especially whether AI benefits vulnerable groups or exacerbates digital and socioeconomic inequalities. Investigating dynamic trade-offs and synergies between AI development and SDG progress will further improve understanding of pathways towards more balanced AI-enabled development.

## 5 Conclusions

This study provided global quantitative evidence of the associations between AI and sustainable development by integrating treatment-effect estimation, mechanism analysis, heterogeneity assessment, and AI–SSP scenario projections. The results demonstrated robust positive associations between AI and SDGs 4, 7, and 9 across the alternative learner configurations. The association with SDG 11 was positive and statistically significant under the RF-based specifications and remained stable across changes in the number of cross-fitting folds; however, it changed sign when alternative base learners were applied, indicating that this finding is model-dependent and should be interpreted with caution. The associations with SDGs 12 and 13 were weaker and less stable across the specifications. Mechanism analysis indicated that human capital accumulation, industrial upgrading, and productivity improvement were the main channels through which AI was associated with SDG progress. Heterogeneity analysis also revealed stronger marginal benefits in developing and low-HDI countries, suggesting that AI development may narrow sustainability gaps when supported by adequate infrastructure and governance capacity. Scenario projections suggested that the SSP1–AI-high pathway offered the most favorable long-term outcome, although regional disparities remained. Overall, AI can contribute to sustainable development, but its benefits depend on coordinated policies that align technological innovation with green transformation, inclusive development, and international cooperation.

## CRediT authorship contribution statement

**Meiling Wu:** Writing – review & editing, Writing – original draft, Visualization, Validation, Software, Methodology, Formal analysis, Data curation, Conceptualization. **Wei Liu:** Writing – review & editing, Writing – original draft, Visualization, Validation, Supervision, Software, Resources, Project administration, Methodology, Funding acquisition, Formal analysis, Data curation, Conceptualization. **Ziqiong He:** Formal analysis, Data curation. **Zhengyang Zhang:** Writing – review & editing, Validation, Supervision, Formal analysis, Data curation.

## Data availability

This study utilized annual data for 69 countries spanning Asia, Europe, North America, South America, Africa, and Oceania from 2000 to 2023. The dataset covers all core variables examined, including indicators of AI development level, core SDGs, control variables, and mechanism variables. Detailed data sources and definitions are provided in the main text and Supplementary Material.

For datasets with minor temporal gaps, missing values were estimated using linear interpolation, provided the missing rate was below 5% to ensure interpolation did not introduce significant bias. All variables were standardized or log-transformed to a unified scale to reduce dimensional differences among indicators.

The underlying data are available from the official United Nations SDG Global Database (https://unstats.un.org/sdgs/) and other authoritative international sources, as cited in the appendices.

## Code availability

The scripts used for data processing, spatial analysis, and figure generation in this study are available from the Supplementary Material. Specifically, the global country-level geographic statistical map was primarily generated using Google Earth Engine (https://earthengine.google.com/). The data visualization and statistical analyses were conducted in R version 4.3.1 (R Core Team, 2023) and Python 3.11. All source code and documentation necessary to reproduce the results of this study can be accessed at https://codeocean.com/capsule/2145695/tree. The figures were drafted in Origin 2024b. The scripts for the main analyses implemented in Google Earth Engine can also be obtained directly from https://earthengine.google.com/.

## Declaration of competing interest

The authors declare that they have no known competing financial interests or personal relationships that could have appeared to influence the work reported in this paper.
