# Supplementary material for: The global diffusion of artificial intelligence exposes an efficiency–transformation gap
Source: Environ Sci Ecotechnol. 2026 Oct 29;34:100784. doi: 10.1016/j.ese.2026.100784 (PMC13769063; doi:10.1016/j.ese.2026.100784)
Supplement: Supplementary file 1 [file mmc1.docx]

**Supplementary Material**

**The global diffusion of artificial intelligence exposes an efficiency–transformation gap**

Meiling Wu ^a^, Wei Liu ^b,*^, Ziqiong He ^a^, Zhengyang Zhang ^c^

^a^ School of Management Science, Chengdu University of Technology, Chengdu 610059, China
^b^ School of Economics and Management, Nanchang University, Nanchang, Jiangxi 330031, China
^c^ Graduate School of Environmental Studies, Tohoku University, Sendai 980-8572, Japan

* Corresponding Author: [xueshuliuwei@ncu.edu.cn](mailto:xueshuliuwei@ncu.edu.cn)(W. Liu).

Summary Information
Number of pages: 23
Figures: S1–S2
Tables: S1–S14
Texts: S1–S3

**Figures**

**Fig. S1 | Comparison of significance scores and prediction errors across different K values**

**Fig. S2 | Global distribution of projected composite SDG scores in 2050 for 69 countries**. Calculated as the mean of core SDGs. The basemap used in a was obtained from China’s official standard map service (http://bzdt.ch.mnr.gov.cn/) and carries map approval number GS (2016) 1663.

**Tables**

**Table S1 |  Framework of the future scenario matrix: annual average change rates (%) of variables under SSPs and AI development scenarios, 2025–2060**

| **Scenario combination** | **GDP** | **Urbanization rate** | **Population** | **Education level** | **Government efficiency** | **AI index** |
| --- | --- | --- | --- | --- | --- | --- |
| **SSP1‑AI‑High** | 3.5 | 1.2 | 0.2 | 2 | 2.2 | 1.6 |
| **SSP1‑AI‑Medium** | 3.5 | 1.2 | 0.2 | 2 | 2.2 | 1.0 |
| **SSP1‑AI‑Low** | 3.5 | 1.2 | 0.2 | 2 | 2.2 | 0.6 |
| **SSP2‑AI‑High** | 2 | 0.9 | 0.5 | 1.2 | 1 | 1.6 |
| **SSP2‑AI‑Medium** | 2 | 0.9 | 0.5 | 1.2 | 1 | 1.0 |
| **SSP2‑AI‑Low** | 2 | 0.9 | 0.5 | 1.2 | 1 | 0.6 |
| **SSP3‑AI‑High** | 0.5 | 0.4 | 0.8 | 0.5 | 0.4 | 1.6 |
| **SSP3‑AI‑Medium** | 0.5 | 0.4 | 0.8 | 0.5 | 0.4 | 1.0 |
| **SSP3‑AI‑Low** | 0.5 | 0.4 | 0.8 | 0.5 | 0.4 | 0.6 |
| **SSP4‑AI‑High** | 2.5 | 0.7 | 0.6 | 1.5 | 0.8 | 1.6 |
| **SSP4‑AI‑Medium** | 2.5 | 0.7 | 0.6 | 1.5 | 0.8 | 1.0 |
| **SSP4‑AI‑Low** | 2.5 | 0.7 | 0.6 | 1.5 | 0.8 | 0.6 |
| **SSP5‑AI‑High** | 4.5 | 1.5 | 0.3 | 1.8 | 1.6 | 1.6 |
| **SSP5‑AI‑Medium** | 4.5 | 1.5 | 0.3 | 1.8 | 1.6 | 1.0 |
| **SSP5‑AI‑Low** | 4.5 | 1.5 | 0.3 | 1.8 | 1.6 | 0.6 |

**Table S2 | Control variables and data sources**

| **Variables** | **Computational definition** | **Interpretation** | **Code name** | **Data source (official database or website)** |
| --- | --- | --- | --- | --- |
| Economic development | GDP per capita (US dollars) | Reflects the level of economic development. | X1 | World Bank, World Development Indicators ([https://data.worldbank.org/indicator/NY.GDP.PCAP.CD](https://data.worldbank.org/indicator/NY.GDP.PCAP.CD)) |
| Population scale | Total population | Reflects population size. | X2 | World Bank, *World Development Indicators* (<https://data.worldbank.org/indicator/SP.POP.TOTL>) |
| Innovation intensity | R&D expenditure (% of GDP) | Indicates investment intensity in technological innovation. | X3 | UNESCO Institute for Statistics ([http://uis.unesco.org](http://uis.unesco.org/)) |
| Education | Gross enrolment ratio (%) | Reflects the level of educational attainment and accessibility. | X4 | UNESCO Institute for Statistics ([http://uis.unesco.org](http://uis.unesco.org/)) |
| Industrial structure | Manufacturing value added (% of GDP) | Represents the level of industrial structural development. | X5 | World Bank, *World Development Indicators* ([https://data.worldbank.org/indicator/NV.IND.MANF.ZS](https://data.worldbank.org/indicator/NV.IND.MANF.ZS)) |
| Energy structure | Share of clean energy in final energy consumption (%) | Reflects the degree of energy decarbonization and cleanliness. | X6 | International Energy Agency ([https://www.iea.org/data-and-statistics](https://www.iea.org/data-and-statistics));  World Bank, *SDG Database* ([https://datacatalog.worldbank.org](https://datacatalog.worldbank.org/)) |
| Environmental pressure | CO₂ emissions per capita (tonnes) | Indicates environmental burden and carbon intensity. | X7 | Global Carbon Atlas ([https://www.globalcarbonatlas.org](https://www.globalcarbonatlas.org/));  World Bank ([https://data.worldbank.org/indicator/EN.ATM.CO2E.PC](https://data.worldbank.org/indicator/EN.ATM.CO2E.PC)) |
| Government efficiency | Governance Index | Reflects the quality and effectiveness of public governance. | X8 | World Bank, *Worldwide Governance Indicators* ([https://info.worldbank.org/governance/wgi](https://info.worldbank.org/governance/wgi)) |

**Table S3 | Evaluation indicator system for AI development level**

| **Primary indicator** | **Secondary indicator** | **Computational formula** | **Attribute** | **Data source** |
| --- | --- | --- | --- | --- |
| **AI development level** | Number of AI patents | Total number of AI‑related patents granted in a given country per year | + | [WIPO (World Intellectual Property Organization, https://www.wipo.int)](https://www.wipo.int/) |
|  | Number of industrial robots | Total stock of industrial robots in a given country at year‑end | + | [IFR (International Federation of Robotics, https://ifr.org)](https://ifr.org/) |
|  | Number of scientific publications in AI | Total number of AI‑related scientific papers published in a given country per year | + | Scopus / Web of Science (https://www.scopus.com / https://www.webofscience.com) |
|  | Secure internet servers per million people | Number of secure internet servers ÷ (total population / 1,000,000) | + | [World Bank Data, (https://data.worldbank.org/indicator/IT.NET.SECR.P6)](World%20Bank%20Data,%20（https://data.worldbank.org/indicator/IT.NET.SECR.P6）) |
|  | R&D technical personnel per million people | Number of R&D technical personnel ÷ (total population / 1,000,000) | + | [UNESCO Institute for Statistics (http://uis.unesco.org)](http://uis.unesco.org/) |
|  | ICT frontier readiness | Based on international ICT development index or ICT‑related sub‑indicators from the Global Competitiveness Report | + | [World Economic Forum – Global Competitiveness Report (https://www.weforum.org/reports)](https://www.weforum.org/reports%EF%BC%89) |

**Table S4 | Core SDG indicator system**

| **SDG** | **Indicator code** | **Indicator name** | **Unit** | **Attribute** | **Data source** |
| --- | --- | --- | --- | --- | --- |
| **SDG 3  (Good health and well‑being)** | 3.1.1 | Maternal mortality ratio (per 100,000 live births) | persons / 100,000 live births | - | United Nations SDG Global Database; WHO Health Statistics (https://unstats.un.org/sdgs ; https://www.who.int/data) |
|  | 3.2.1 | Under‑5 mortality rate (per 1,000 live births) | persons / 1,000 live births | - | [WHO Global Health Observatory (https://www.who.int/data/gho)](https://www.who.int/data/gho) |
|  | 3.8.1 | Coverage index of essential health services | index (0–100) | + | [WHO; World Bank Data (https://data.worldbank.org)](https://data.worldbank.org/) |
| **SDG 4  (Quality education)** | 4.1.2 | Youth and adult literacy rate | % | + | [UNESCO Institute for Statistics (https://uis.unesco.org)](https://uis.unesco.org/) |
|  | 4.3.1 | Share of population receiving lifelong learning training (aged 15–64) | % | + | UNESCO UIS |
|  | 4.5.1 | Gender parity index in education | index | + | UN Data / World Bank |
| **SDG 7  (Affordable and clean energy)** | 7.1.2 | Population with access to electricity | % of total population | + | [World Bank – SE4ALL Database (https://data.worldbank.org/indicator/EG.ELC.ACCS.ZS)](https://data.worldbank.org/indicator/EG.ELC.ACCS.ZS) |
|  | 7.2.1 | Renewable energy share in final energy consumption | % | + | [IEA & UN SDG Database (https://unstats.un.org/sdgs/metadata)](https://unstats.un.org/sdgs/metadata) |
|  | 7.3.1 | Energy intensity (energy use per unit GDP) | megajoules / USD | - | [IEA Statistics (https://www.iea.org/data-and-statistics)](https://www.iea.org/data-and-statistics) |
| **SDG 9 (Industry, innovation and infrastructure)** | 9.2.1 | Manufacturing value added as share of GDP | % | + | [World Bank (https://data.worldbank.org)](https://data.worldbank.org/) |
|  | 9.4.1 | CO₂ emissions per unit of value added | kg CO₂ / USD | - | UNFCCC / IEA |
|  | 9.5.1 | R&D expenditure as share of GDP | % | + | UNESCO UIS |
|  | 9.5.2 | R&D personnel per million people | persons / million population | + | UNESCO UIS |
| **SDG 11  (Sustainable cities and communities)** | 11.1.1 | Proportion of urban population living in slums | % of urban population | - | [UN‑Habitat (https://data.unhabitat.org)](https://data.unhabitat.org/) |
|  | 11.6.1 | Urban air quality (PM₂․₅ concentration) | μg / m³ | - | WHO Air Quality Database |
|  | 11.6.2 | Proportion of municipal solid waste that is managed | % | + | UN‑Habitat |
| **SDG 12  (Responsible consumption and production)** | 12.2.2 | Resource productivity (GDP per material consumption) | USD / kg | + | [UNEP (https://environmentlive.unep.org)](https://environmentlive.unep.org/) |
|  | 12.4.2 | Total hazardous waste generated | tonnes / year | - | UNEP Chemicals and Waste |
|  | 12.5.1 | Material recycling rate (share of recycled material in total waste) | % | + | OECD / UNEP |
| **SDG 13 (Climate action)** | 13.2.2 | Per capita CO₂ emissions | tonnes / person·year | - | [Global Carbon Atlas / UNFCCC (https://www.globalcarbonatlas.org)](https://www.globalcarbonatlas.org/) |
|  | 13.3.1 | National‑level climate education and awareness programmes | index (0–1) | + | UN Environment Programme |
|  | 13.2.1 | Number of climate actions integrated into national policies or strategies | count / country | + | UN SDG Global Database |

**Table S5| Descriptive statistics of all variables.**

|  | **Count** | **Mean** | **Std** | **Min** | **25%** | **50%** | **75%** | **Max** |
| --- | --- | --- | --- | --- | --- | --- | --- | --- |
| **SDG3_score** | 1656 | 60.99457 | 25.53468 | 0 | 42.2425 | 62.19035 | 83.21382 | 100 |
| **SDG4_score** | 1656 | 49.37913 | 8.079946 | 23.434 | 45.03186 | 50.42824 | 54.95296 | 69.69025 |
| **SDG7_score** | 1656 | 63.21041 | 10.02681 | 39.8362 | 55.79826 | 63.86551 | 69.98914 | 90.97458 |
| **SDG9_score** | 1656 | 78.7041 | 5.502693 | 60.88222 | 75.01059 | 78.6431 | 82.33934 | 90.71511 |
| **SDG11_score** | 1656 | 77.96222 | 14.2919 | 27.44756 | 68.10879 | 82.27483 | 89.48264 | 97.71355 |
| **SDG12_score** | 1656 | 41.21964 | 6.809635 | 20.3447 | 36.53838 | 40.50509 | 46.24688 | 72.64597 |
| **SDG13_score** | 1656 | 75.56225 | 30.73108 | 1.84696 | 52.69693 | 100 | 100 | 100 |
| **AI_score** | 1656 | 0.287606 | 0.260045 | 0 | 0.077706 | 0.195615 | 0.433991 | 0.995098 |
| **X1** | 1656 | 23554.39 | 22387.51 | 381.409 | 5190.454 | 15680.58 | 38321.37 | 108798.5 |
| **X2** | 1656 | 78639832 | 2.21E+08 | 281205 | 5166465 | 11508827 | 63200343 | 1.44E+09 |
| **X3** | 1656 | 1.185913 | 1.003053 | 0.0159 | 0.442535 | 0.8284 | 1.718615 | 6.01924 |
| **X4** | 1655 | 57.24808 | 25.50912 | 2.38838 | 37.93538 | 59.84876 | 74.64444 | 166.6656 |
| **X5** | 1656 | 15.86654 | 6.559299 | 4.425565 | 11.247 | 14.9267 | 18.82328 | 48.44288 |
| **X6** | 1656 | 11.56261 | 14.62389 | 0 | 1.335 | 6.775 | 15.4725 | 89.72 |
| **X7** | 1656 | 483.4142 | 736.2064 | 0.053298 | 76.51509 | 242.9089 | 623.463 | 6034.564 |
| **X8** | 1656 | 0.777084 | 1.325683 | -1.225 | -0.1195 | 0.6145 | 1.45025 | 8 |

**Table S6 | Parameter Settings of Eleven Comparative Models.**

| **Model** | **Hyperparameters** |
| --- | --- |
| LightGBM | n_estimators=300; learning_rate=0.05; max_depth=-1; random_state=42 |
| XGBoost | n_estimators=300; learning_rate=0.05; max_depth=6; subsample=0.8; colsample_bytree=0.8; objective=reg:squarederror; random_state=42 |
| RandomForest | n_estimators=300; max_depth=10; min_samples_leaf=2; random_state=42 |
| GradientBoosting | n_estimators=300; learning_rate=0.05; max_depth=4; random_state=42 |
| NeuralNetwork | hidden_layer_sizes=(100,50); activation=relu; solver=adam; max_iter=2000; random_state=42 |
| KNN | n_neighbors=5; weights=distance |
| SVM | kernel=rbf; C=10; gamma=scale |
| LinearRegression | default |
| Ridge | alpha=1.0; random_state=42 |
| Lasso | alpha=0.01; random_state=42; max_iter=10000 |
| Voting_SVM_NN | VotingRegressor with SVM and NeuralNetwork; equal weights |

**Table S7 | Comparison of model performance and cross-task variability across SDG outcomes (model_avg_metrics)**

| Model | MAE_mean | RMSE_mean | R2_mean | MAE_std | RMSE_std | R2_std | N_Tasks | R2_norm | MAE_rev_norm | RMSE_rev_norm | Final_Score |
| --- | --- | --- | --- | --- | --- | --- | --- | --- | --- | --- | --- |
| LightGBM | 6.09 | 8.40 | 0.60 | 5.02 | 6.58 | 0.27 | 7 | 0.96 | 1.00 | 1.00 | 0.99 |
| RandomForest | 6.32 | 8.56 | 0.60 | 4.51 | 6.06 | 0.20 | 7 | 0.98 | 0.96 | 0.97 | 0.97 |
| XGBoost | 6.58 | 8.62 | 0.62 | 5.29 | 6.66 | 0.20 | 7 | 1.00 | 0.91 | 0.96 | 0.96 |
| GradientBoosting | 6.43 | 8.54 | 0.59 | 5.06 | 6.54 | 0.24 | 7 | 0.96 | 0.94 | 0.97 | 0.96 |
| Voting_SVM_NN | 8.11 | 10.49 | 0.45 | 6.21 | 8.00 | 0.21 | 7 | 0.71 | 0.63 | 0.64 | 0.66 |
| KNN | 8.94 | 11.48 | 0.36 | 6.89 | 8.76 | 0.16 | 7 | 0.55 | 0.48 | 0.47 | 0.50 |
| SVM | 9.33 | 11.87 | 0.31 | 7.24 | 9.00 | 0.19 | 7 | 0.46 | 0.41 | 0.40 | 0.42 |
| NeuralNetwork | 9.04 | 12.48 | 0.18 | 7.17 | 10.45 | 0.57 | 7 | 0.24 | 0.46 | 0.30 | 0.33 |
| Lasso | 11.58 | 14.19 | 0.05 | 10.35 | 12.04 | 0.59 | 7 | 0.00 | 0.00 | 0.00 | 0.00 |
| Ridge | 11.58 | 14.20 | 0.05 | 10.35 | 12.03 | 0.59 | 7 | 0.00 | 0.00 | 0.00 | 0.00 |
| LinearRegression | 11.59 | 14.21 | 0.05 | 10.36 | 12.04 | 0.59 | 7 | 0.00 | 0.00 | 0.00 | 0.00 |

Note: This table provides the underlying data for Fig. 3m-p. All models were evaluated across the seven SDG outcomes using the same dataset, train-test split, preprocessing pipeline, random seed, and evaluation protocol. MAE_mean, RMSE_mean, and R²_mean report the cross-outcome average predictive errors and explanatory power, and MAE_std, RMSE_std, and R²_std report the corresponding cross-outcome variability. R2_norm, MAE_rev_norm, and RMSE_rev_norm are normalized metrics used to calculate the comprehensive performance score, with reverse normalization applied to MAE and RMSE so that higher values indicate better performance. Final_Score is the mean of the three normalized indicators and summarizes overall model performance. Model configurations are provided in Table S13.

**Table S8 | Results of benchmark regression**

| **Variables** | **Artificial intelligence** | | | |
| --- | --- | --- | --- | --- |
|  | **(1)** | **(2)** | **(3)** | **(4)** |
|  | **Country FE** | **Country & Year FE** | **Controls & Country FE** | **Controls & Country & Year FE** |
| **SDG11** | 8.658*** | 11.946*** | 3.785** | 4.054*** |
|  | (1.054) | (1.844) | (1.558) | (1.545) |
| **SDG12** | 3.733*** | 1.429* | 0.410 | -0.477 |
|  | (0.544) | (0.846) | (0.690) | (0.706) |
| **SDG13** | -0.922 | -1.229 | -3.047 | -3.541 |
|  | (2.320) | (3.947) | (2.353) | (2.525) |
| **SDG3** | 15.572*** | 30.739*** | 5.587 | 7.846** |
|  | (2.093) | (3.666) | (3.570) | (3.586) |
| **SDG4** | 8.743*** | 9.373*** | 3.314*** | 4.050*** |
|  | (0.584) | (1.148) | (0.822) | (0.856) |
| **SDG7** | 12.030*** | 13.738*** | 2.824** | 3.745*** |
|  | (0.739) | (1.387) | (1.178) | (1.184) |
| **SDG9** | 2.337*** | 3.402*** | 0.859** | 0.994** |
|  | (0.440) | (0.727) | (0.413) | (0.424) |
| **K-folds** | 6 | 6 | 6 | 6 |
| **Base learner** | RF | RF | RF | RF |

Note 1: Parentheses indicate the clustered standard errors. *, **, *** indicate statistical significance at the 10 %, 5 %, and 1 % levels, respectively. The same below.

Note 2: Consistent with the multidimensional nature of both constructs, the AI development index and the SDG9 indicator system incorporate a few shared R&D-related indicators (Table S12). Given that the AI index is aggregated from six indicators using entropy-based weights, this compositional feature does not affect the estimated associations for the other six SDGs.

**Table S9 | Robustness test: resetting K-folds value and base learner**

| **Outcome** | **(1)** | **(2)** | **(3)** | **(4)** | **(5)** |
| --- | --- | --- | --- | --- | --- |
|  | **RF, K=5** | **RF, K=7** | **LightGBM, K=6** | **XGBoost, K=6** | **Ridge, K=6** |
| **SDG3** | 9.445*** | 10.241*** | -4.496 | -5.811* | -1.047 |
|  | (3.579) | (3.611) | (3.643) | (3.376) | (4.574) |
| **SDG4** | 3.917*** | 4.120*** | 2.587*** | 2.238*** | 3.521*** |
|  | (0.845) | (0.861) | (0.714) | (0.652) | (0.754) |
| **SDG7** | 3.073*** | 3.315*** | 3.596*** | 1.681** | 4.380*** |
|  | (1.190) | (1.198) | (0.859) | (0.676) | (0.787) |
| **SDG9** | 1.074** | 1.050** | 0.785* | 0.440 | 1.250*** |
|  | (0.431) | (0.436) | (0.415) | (0.439) | (0.484) |
| **SDG11** | 4.047*** | 4.409*** | -0.901 | -1.015 | -5.535*** |
|  | (1.517) | (1.562) | (1.521) | (1.317) | (1.364) |
| **SDG12** | -0.706 | -0.597 | 0.574 | 0.776 | 3.265*** |
|  | (0.704) | (0.728) | (0.665) | (0.625) | (0.770) |
| **SDG13** | -2.404 | -1.270 | 0.416 | 0.765 | 0.133 |
|  | (2.409) | (2.417) | (1.360) | (1.133) | (1.680) |

**Table S10 | Mechanism variables and data sources**

| **Variables** | **Computational definition** | **Interpretation** | **Data source (official database or website)** |
| --- | --- | --- | --- |
| Human capital level | Gross tertiary enrolment ratio (% of total population) | Reflects national human capital accumulation and the level of higher‑education participation | [UNESCO Institute for Statistics — Gross tertiary enrolment ratio (https://uis.unesco.org)](https://uis.unesco.org/) |
| Green innovation | Number of green patent applications | Indicates capacity for green technological innovation and environmentally friendly R&D output | WIPO Green & PATSTAT — World Intellectual Property Organization (https://www3.wipo.int/ipstats/);  OECD Patent Database (https://stats.oecd.org) |
| Government efficiency | Worldwide Governance Indicators (WGI score) | Measures quality of governance and efficiency of public‑service delivery | [World Bank — Worldwide Governance Indicators (https://info.worldbank.org/governance/wgi)](https://info.worldbank.org/governance/wgi) |
| Advanced industrial structure | Service‑sector value added (% of GDP) | Assesses the share of the tertiary sector, reflecting industrial upgrading towards higher‑value activities | [World Bank — World Development Indicators (https://data.worldbank.org/indicator/NV.SRV.TETC.ZS)](https://data.worldbank.org/indicator/NV.SRV.TETC.ZS) |
| Total factor productivity | Total factor productivity (TFP) | Captures efficiency in the combined use of capital and labour | [Penn World Table (PWT 10.0) — Groningen Growth and Development Centre](https://www.rug.nl/ggdc/productivity/pwt/) *[(https://www.rug.nl/ggdc/productivity/pwt/)](https://www.rug.nl/ggdc/productivity/pwt/)* |
| Energy efficiency | Energy use per US$ 1,000 GDP (kg oil equivalent, 2021 constant PPP prices) | Measures efficiency in energy use and progress towards green development | International Energy Agency — Energy Intensity Indicators (https://www.iea.org/data-and-statistics); World Bank (https://data.worldbank.org/indicator/EG.EGY.PRIM.PP.KD) |

Note: Energy efficiency is proxied by energy intensity (energy use per US$1,000 GDP); a lower value indicates higher efficiency.

**Table S11 | DML-Based mechanism analysis of AI’s impact on mechanism variables**

| **Variables** | **Human capital** | **Green innovation** | **Government efficiency** | **Industrial structure** | **TFP** | **Energy efficiency** |
| --- | --- | --- | --- | --- | --- | --- |
| **AI** | 0.010*** | -0.010*** | -0.001*** | 0.021*** | 0.004*** | -0.040*** |
|  | (0.001) | (0.001) | (0.000) | (0.001) | (0.001) | (0.002) |
| **Controls** | YES | YES | YES | YES | YES | YES |
| **Country** | YES | YES | YES | YES | YES | YES |
| **Year** | YES | YES | YES | YES | YES | YES |
| **Countries** | 69 | 69 | 69 | 69 | 69 | 69 |
| **Observations** | 1656 | 1656 | 1656 | 1656 | 1656 | 1656 |
| **K-folds** | 6 | 6 | 6 | 6 | 6 | 6 |
| **Base learner** | RF | RF | RF | RF | RF | RF |

**Table S12 | Mechanism-based analysis of mechanisms’ effects on SDGs (DML, RF, K = 6)**

| **Variables** | **Education** | **Green innovation** | **Government efficiency** | **Industrial structure** | **TFP** | **Energy efficiency** |
| --- | --- | --- | --- | --- | --- | --- |
| **SDG3** | -0.0226 *** | 0.1576 *** | 0.0306 *** | -0.0466 *** | -0.0357 *** | 0.0377 *** |
|  | (-0.0028) | -0.0124 | -0.0047 | -0.0084 | -0.0042 | -0.0074 |
| **SDG4** | 0.0332 *** | 0.0360 *** | 0.2188 *** | 0.1419 *** | -0.0447 *** | -0.0906 *** |
|  | -0.0044 | -0.0071 | -0.0255 | -0.0106 | -0.0135 | -0.0054 |
| **SDG7** | -0.0178 *** | -0.0539 *** | 0.1730 *** | 0.1845 *** | 0.2239 *** | -0.7178 *** |
|  | -0.0049 | -0.0048 | -0.013 | -0.0178 | -0.0097 | -0.0198 |
| **SDG9** | -0.0678 *** | 0.1048 *** | 0.0311 *** | -0.0128 *** | 0.0178 *** | -0.0165 *** |
|  | -0.0065 | -0.0107 | -0.0059 | -0.0031 | -0.0035 | -0.0039 |
| **SDG11** | -0.0405 *** | -0.0386 *** | 0.2219 *** | 0.1908 *** | 0.0220 *** | -0.1198 *** |
|  | -0.0058 | -0.0046 | -0.0153 | -0.0055 | -0.0043 | -0.0061 |
| **SDG12** | -0.0768 *** | 0.0726 *** | 0.0430 *** | 0.0211 *** | 0.3934 *** | 0.1034 *** |
|  | -0.0126 | -0.012 | -0.0043 | -0.0067 | -0.0171 | -0.0093 |
| **SDG13** | -0.0213 *** | 0.1457 *** | -0.0008 | -0.0341 *** | -0.0627 *** | 0.0072 * |
|  | -0.0022 | -0.0102 | -0.0007 | -0.005 | -0.0034 | -0.004 |
| **Controls** | YES | YES | YES | YES | YES | YES |
| **Country FE** | YES | YES | YES | YES | YES | YES |
| **Year FE** | YES | YES | YES | YES | YES | YES |
| **Countries** | 69 | 69 | 69 | 69 | 69 | 69 |
| **Observations** | 1 656 | 1 656 | 1 656 | 1 656 | 1 656 | 1 656 |
| **K‑folds** | 6 | 6 | 6 | 6 | 6 | 6 |
| **Base learner** | RF | RF | RF | RF | RF | RF |

Note: Values represent the average marginal effect of a one‑standard‑deviation increase in the mechanism variable on the corresponding SDG indicator. Significance levels: *** p < 0.01; ** p < 0.05; * p < 0.10.

**Table S13 | Heterogeneity analysis results**

| **Variables** | **High HDI** | **Low HDI** | **Developed countries** | **Developing countries** |
| --- | --- | --- | --- | --- |
| **SDG3** | -3.6029 | -10.3432 | -5.5866 | 0.4342 |
|  | (2.7274) | (15.9506) | (3.5144) | (4.0483) |
| **SDG4** | 1.2937** | 11.6253*** | 1.6681** | 2.1965*** |
|  | (0.5068) | (3.4962) | (0.7240) | (0.6934) |
| **SDG7** | 0.6423 | 6.1697** | 2.8053*** | -0.4735 |
|  | (0.5801) | (2.7727) | (0.6162) | (0.7994) |
| **SDG9** | 0.4750 | -4.4606 | 0.8478** | 0.9357 |
|  | (0.4086) | (4.5015) | (0.3709) | (0.6674) |
| **SDG11** | 0.2055 | -1.3134 | 0.8399 | -1.4113 |
|  | (1.2121) | (7.3751) | (1.6269) | (1.3979) |
| **SDG12** | 0.2874 | 4.1814* | -0.0840 | 1.9096*** |
|  | (0.5274) | (2.4387) | (0.7007) | (0.6920) |
| **SDG13** | -0.1671 | 4.4506 | -2.2601 | 0.1615 |
|  | (1.0545) | (4.9061) | (1.6528) | (1.5255) |
| **Controls** | YES | YES | YES | YES |
| **Country fixed effects** | YES | YES | YES | YES |
| **Year fixed effects** | YES | YES | YES | YES |
| **Base learner** | RF | RF | RF | RF |
| **K-folds** | 6 | 6 | 6 | 6 |
| **Observations** | 1584 | 72 | 792 | 864 |
| **Countries** | 66 | 3 | 33 | 36 |

Note: In the HDI-based grouping, countries were classified as low-HDI when their HDI in the baseline year 2000 (the first year of the sample) fell below the UNDP threshold of 0.550 [UNDP, Human Development Report], identifying exactly three countries—India (HDI2000 = 0.498), Morocco (0.529), and Pakistan (0.481)—whereas the remaining 66 countries exhibit HDI2000 ≥ 0.550. The subsample is small because our sample-selection criteria require complete data on the AI, SDG, and control indicators (Tables S11 and S12), which excludes most very-low-HDI economies, particularly in Sub-Saharan Africa, owing to missing data on AI-related indicators. The three countries span South Asia and North Africa and together account for approximately one-fifth of the global population.

**Table S14 | Leave-one-country-out sensitivity analysis for the low-HDI group.**

| **Variables** | **Full** | **Excluding India** | **Excluding Morocco** | **Excluding Pakistan** |
| --- | --- | --- | --- | --- |
| **SDG3** | -12.4161 | -24.8855 | 8.0587 | -2.8841 |
|  | (16.0972) | (18.8690) | (15.2362) | (16.6949) |
| **SDG4** | 11.3110*** | 7.8364 | 8.7242*** | 8.2658*** |
|  | (3.5223) | (5.8578) | (2.4255) | (2.5758) |
| **SDG7** | 6.2568** | 1.3461 | 9.1262*** | 4.8902 |
|  | (2.7949) | (3.5071) | (2.4111) | (3.3091) |
| **SDG9** | -4.6323 | -0.4823 | -2.2321 | -2.2020 |
|  | (4.5613) | (3.2702) | (3.3655) | (2.9956) |
| **SDG11** | -0.9947 | -3.6366 | -0.0749 | 2.8033 |
|  | (7.3641) | (8.1939) | (6.0979) | (7.6799) |
| **SDG12** | 4.0705* | 3.1587 | 17.6244*** | 17.2575*** |
|  | (2.4238) | (2.2146) | (3.6722) | (2.8978) |
| **SDG13** | 4.2240 | 3.8504 | n/a† | 3.3055 |
|  | (4.9530) | (8.9771) | – | (6.5951) |
| **Observations** | 72 | 48 | 48 | 48 |
| **Countries** | 3 | 2 | 2 | 2 |

Note: Standard errors are in parentheses. ***, **, and * denote statistical significance at the 1%, 5%, and 10% levels, respectively. Each column re-estimates the DML model—Random Forest base learners with K = 6-fold cross-fitting, the control variables, and country and year fixed effects—after excluding the indicated country, such that two countries (48 observations) remain.

**Texts**

**Text S1. Model Optimization, Validation, and Scenario Projection Procedure of the QRCNN–LSTM–Transformer**

This section describes the optimization, validation, and application procedures of the QRCNN–LSTM–Transformer model, including data partitioning, look-back window selection, structural parameter adjustment, hyperparameter tuning, and the recursive scenario-based projection procedure used for long-term SDG simulations：

**(1) Data partitioning and validation framework**

The model was developed using the annual dataset for 2000–2023. To evaluate out-of-sample predictive performance, the data were divided into a training period and a validation period. Observations from 2000 to 2018 were used for model training, while the model was validated against the observed values for 2019–2023. Because sequence-based prediction requires preceding historical observations as model input, the validation samples for 2019–2023 were generated from rolling historical sequences extending back to 2014 under different candidate look-back settings. This design ensured that model selection was based on temporal generalization performance rather than in-sample fit alone.

**(2) Look-back window optimization**

The first step of model optimization was to determine the appropriate look-back window length, i.e., the number of preceding annual observations used as input for each one-step-ahead prediction. Candidate window lengths of 4, 5, 6, and 7 years were compared. The normalized root mean square error (RMSE_norm) values for these candidate windows were 0.2058, 0.1937, 0.1938, and 0.1928, respectively. Although the seven-year window produced a slightly lower RMSE_norm, comprehensive evaluation across RMSE, MAE, and MAPE indicated that the five-year window achieved the best overall validation performance (RMSE_norm = 0.1937, MAE_norm = 0.1524, RMSE_orig = 15.38, MAE_orig = 10.63, MAPE_orig = 29.72%). Therefore, the optimal look-back window length was set to 5.

It should be emphasized that the look-back window refers only to the length of the historical input sequence used at each prediction step. A five-year look-back means that the model uses the preceding five annual observations to predict the next-year SDG outcome. It does not mean that the model was trained using only five years of data, nor that the long-term trajectories to 2100 were directly extrapolated from a single five-year sequence. Instead, the model was trained on the full historical dataset within the training period, and the five-year window was selected as the optimal input length for sequential prediction.

**(3) Structure and hyperparameter optimization**

After fixing the look-back window at 5, the network structure and training hyperparameters were systematically optimized. For the recurrent component, three configurations were evaluated: LSTM (64, 32), LSTM (128, 64), and BiLSTM (64, 32). Performance differences among these alternatives were minimal across varying noise levels (RMSE difference < 0.07), and the more complex architectures did not yield significant gains in predictive accuracy or robustness. Therefore, the two-layer LSTM (64, 32) structure was retained because it provided the best balance between model simplicity and forecasting effectiveness.

Hyperparameter tuning was then conducted over the following ranges: learning rate (0.0002–0.001), batch size (8–32), optimizer (Adam, AdamW, Nadam), and number of training epochs (100–200), in conjunction with an EarlyStopping mechanism to reduce overfitting. Validation results for 2019–2023 showed that the Nadam optimizer achieved the best performance, with RMSE_norm = 0.1787 and MAE_norm = 0.1149, outperforming both Adam and AdamW. Based on a comprehensive assessment of validation accuracy, convergence behavior, and generalization stability, the optimal training configuration was determined as follows: learning rate = 0.001, batch size = 8, epochs = 100, with EarlyStopping enabled.

**(4) Optimal model configuration and validation performance**

Based on the minimum RMSE_norm criterion and the overall validation performance, the final hyperparameter configuration was determined as follows: CNN_filters = 32; CNN_kernel = 2; Transformer_heads = 4; ff_dim = 64; Dropout = 0.0; Optimizer = Nadam; Learning_rate = 0.001; Batch_size = 8; Epochs = 100 (with EarlyStopping); Lookback = 5.

Under this configuration, the model achieved the following validation performance: RMSE_norm = 0.0470 and MAE_norm = 0.0298. On the original scale, the corresponding evaluation metrics were MAE_orig = 0.0298 and MAPE_orig = 25.7%. These results indicate that the selected architecture provided strong predictive accuracy and stable generalization performance for multi-scenario SDG time-series modelling.

**(5) Final model architecture**

The final QRCNN–LSTM–Transformer architecture consisted of a Conv1D layer with 32 filters and a kernel size of 2, followed by a MaxPooling1D layer for local feature extraction and dimensionality reduction. The extracted features were then passed through two stacked LSTM layers with 64 and 32 units, respectively, to learn sequential temporal dependencies from the historical observations. Next, a Transformer encoder with 4 attention heads and a feed-forward dimension of 64 was applied to capture broader temporal interactions and cross-scenario dependencies. Finally, a Dense output layer with a linear activation function was used to generate the SDG projection for the next time step.

This architecture enabled the model to integrate local pattern extraction, sequential memory, and global dependency learning within a unified framework, thereby providing effective error control and high stability in long-term multi-scenario SDG projection tasks.

**(6) Recursive scenario-based projection procedure**

After the optimal model structure and parameter settings were established, the trained model was used to generate SDG projections for 2024–2100 under the SSP–AI scenario combinations. In each projection step, the model took the most recent five-year sequence of relevant variables as input, including both SDG-related historical information and scenario-specific driver trajectories. The model then produced the SDG value for the next year. This newly generated output was subsequently incorporated into the input sequence for the following prediction step, together with the corresponding SSP–AI scenario inputs, thereby forming a recursive year-by-year projection process until 2100.

Accordingly, the long-term SDG trajectories presented in the main text were not obtained by directly extrapolating a 75-year horizon from only five observed years. Rather, they were derived from a recursive scenario-based simulation framework trained on the full historical dataset and driven by externally specified SSP–AI pathways. This procedure allowed the model to extend historically learned temporal relationships into internally consistent long-term scenario trajectories, while acknowledging that uncertainty may increase over longer projection horizons.

**Text S2. Calculation of comprehensive score -- Entropy Weight Method.**

In this study, the entropy method is used to calculate the AI development level and each core SDGs score. The calculation process is as follows:

(1) Standardized indicators data.

Positive indicators:

$x_{ij}=\frac{x_{ij}-min\left( x_{j} \right)}{max\left( x_{j} \right)-\min\left( x_{j} \right)}\left( i=1,2,\cdots,m;j=1,2,\cdots,n \right)$	(1)

Negative indicators:

$x_{ij}=\frac{max\left( x_{j} \right)-x_{ij}}{max\left( x_{j} \right)-min \left( x_{j} \right)}\left( i=1,2,\cdots,m;j=1,2,\cdots,n \right)$	(2)

(2) Calculation of entropy-based normalized decision matrix.

$p_{ij}=\frac{x_{ij}}{\sum_{i=1}^{m} x_{ij}}\left( i=1,2,\cdots,m;j=1,2,\cdots,n \right)$	(3)

(3) Calculation of entropy values.

$e_{j}=-k\sum_{j=1}^{n} p_{ij}lnp_{ij}$	(4)

where $k=\frac{1}{ln(m)}$.

(4) Calculation of weights for each indicator.

$w_{j}=\frac{1-e_{j}}{\sum_{j=1}^{n} d_{j}}$	(5)

(5) Calculate the comprehensive score.
$S_{i}=\sum_{j=1}^{n} w_{j}x_{ij}$	(6)

**Text S3. Setup procedure of the Double Machine Learning (DML) approach.**

To ensure the robustness of the DML model’s predictive performance on long-term panel data, this study systematically assesses the fitting accuracy and generalization ability of 11 commonly used supervised learning algorithms. The selected models represent a broad spectrum of machine learning techniques, including ensemble methods (Random Forest, Gradient Boosting, XGBoost, LightGBM, and Voting_SVM_NN), linear regression approaches (Linear Regression, Ridge, and Lasso), a nonparametric algorithm (KNN), support vector machine (SVM), and a neural network (Neural Network). By conducting multiple rounds of cross-validation across AI and seven core SDG-related indicators, the average test-set performance metrics for each model are calculated, specifically Mean Absolute Error (MAE), Root Mean Squared Error (RMSE), and the coefficient of determination (R²). A normalized combination of these three metrics is used to compute an integrated evaluation score—referred to as performance_mean—to enable effective model comparison. Aggregated results across multiple indicators (see Table S2 and Figure 3) demonstrate that the Random Forest algorithm yields the best overall performance under the multi-dimensional assessment criteria. Consequently, Random Forest is adopted as the primary prediction model in the first stage of the DML framework. To enhance the unbiasedness of DML estimation, a cross-fitting strategy is implemented to reduce overfitting risks arising from high-dimensional covariates. Specifically, the entire sample is randomly divided into K disjoint subsets {S₁, S₂, ..., Sₖ}, with each subset sequentially serving as a validation set while the remaining (K−1) subsets form the corresponding training set S₋ₖ. Within each training set, Random Forest (RF) is applied as the base estimator to separately predict g(Xi,T) and m(Xi,T), where Xi comprises eight confounding variables.
